# Spatial information matters: are traditional imputation methods effective for spatial transcriptomics data?

**DOI:** 10.1093/bib/bbag027

**Published:** 2026-02-02

**Authors:** Fahim Hafiz, Riasat Azim, Swakkhar Shatabda

**Affiliations:** Department of Computer Science and Engineering, United International University, Madani Avenue, Dhaka-1212, Bangladesh; Department of Computer Science and Engineering, United International University, Madani Avenue, Dhaka-1212, Bangladesh; Department of Computer Science and Engineering, Brac University, Pragati Sarani, Dhaka-1212, Bangladesh

**Keywords:** spatially resolved transcriptomics, single-cell RNA, dropout imputation, spatial information, deep learning

## Abstract

Recent advancements in spatially resolved transcriptomics (SRT) have enabled near single-cell resolution, providing rich spatial context crucial for uncovering biological insights. However, high-resolution SRT datasets remain sparse and prone to dropout events that may impede accurate interpretation. Computational imputation methods are often employed to recover missing values, yet existing state-of-the-art (SOTA) techniques—designed for tabular, single-cell RNA, or general SRT data—have not been systematically benchmarked on datasets produced by newer SRT technologies. In this study, we evaluate seven SOTA imputation methods across five emerging SRT platforms encompassing 23 datasets. Our results reveal that no single method consistently excels, with most struggling to accurately identify valid dropouts. Motivated by these limitations, we introduce `SpaMean-Impute', a novel imputation method tailored for SRT datasets that incorporates spatial information to mitigate dropout effects and detect valid dropouts. Our proposed method outperforms the SOTA imputation methods across evaluation metrics, such as adjusted rand index (ARI), normalized mutual information (NMI), adjusted mutual information (AMI), and homogeneity (HOMO). In case of ARI, the proposed method outperforms the SOTA methods on average 16.15%, whereas 18.45% improvement in NMI, 18.96% in AMI, and 13.98% in the case of HOMO. Furthermore, the proposed method is computationally efficient compared with other SOTA methods. For example, compared with the SOTA deep-learning-based imputation methods, the proposed method is $\sim 33\times $ faster and requires, on average, 1500 MB less memory during imputation. Moreover, our approach offers notable computational efficiency. Source code, datasets, and benchmarking scripts are available at: https://github.com/FahimHafiz/SpaMean-Impute.

## Introduction

Transcriptomics is the study of RNA molecules (i.e. transcripts) expressed in cells, tissues, or organisms, unveiling gene activity as well as its role in biological processes and disease progression in the living body [1, 2]. Tools like RNA sequencing (RNA-seq) analyze gene expression, recognizing patterns as well as cellular states in the tissue. In transcriptomic studies, bulk RNA-seq provides averaged gene expression from the tissue sample while elucidating global gene expression patterns and disease-specific markers [2]. However, bulk RNA-seq fails to provide accurate cell-specific functions, cellular heterogeneity, as well as spatial context among the cells [2, 3]. Conversely, single-cell RNA-seq (scRNA-seq) isolates single cells, providing intercellular heterogeneity and functionalities at the single-cell resolution, but loses overall spatial context among cells, which results in limited insights on intercellular interactions [4]. Spatially resolved transcriptomics (SRT) technology resolves such limitations of bulk RNA-seq and scRNA-seq by preserving the spatial relationship among the cells while providing gene expression, making it ideal for understanding how cells interact, tissue structures, disease mechanisms, and treatment strategies for the affected organisms [1, 2, 5]. SRT even allows certain reconstruction of 3D tissue architecture directly from 2D slices [6]. In this way, SRT retains crucial details about cellular heterogeneity and the spatial organization of tissues, enabling a deeper understanding of cellular interactions, functional states, and microenvironmental interactions [2, 7–9]. For example, in developmental biology, SRT allows the investigation of cellular interactions and symmetry breaking during tissue development [7], while in clinical applications, it helps uncover abnormal spatial organization in disease states such as cancer that is critical for diagnosis and therapy selection [2]. Moreover, the power of SRT technologies in deciphering tissue complexity has enabled the generation of atlases of critical biological processes, such as tissue development or organoid formation [10, 11], and has provided insights into human disease pathogenesis [12, 13]. Despite the rapid technological progress, the field remains in its early developmental stages, with ongoing efforts like the SpaceTX consortium aiming to standardize and benchmark imaging-based spatial transcriptomics methods [8, 9]. SRT technologies are generally divided into two main categories: sequencing-based methods and imaging-based methods [2, 8, 9, 14, 15]. Sequencing-based methods, such as 10$\times $ Genomics Visium, Slide-seqV2, and Stereo-seq, use next-generation sequencing technologies to capture polyadenylated RNA from tissue samples, offering varying resolutions from tissue-wide to near-single-cell scales [7]. These techniques provide unbiased whole-transcriptome analysis, ideal for discovering novel biological mechanisms [7]. In contrast, imaging-based methods, including MERFISH, seqFISH, and STARmap, rely on *in situ* hybridization and advanced imaging to capture transcript data with subcellular resolution, making them particularly useful for studying intracellular organization and molecular interactions [2, 7, 14]. An extended classification further distinguishes between imaging-based, sequencing-based (microdissection-based), *in situ* sequencing-based, and *in situ* spatial barcoding-based approaches [9]. Imaging-based methods (e.g. smFISH, RNAscope, seqFISH, and MERFISH) use *in situ* hybridization to target RNA molecules in cells, tissue sections, or FFPE samples [9]. Sequencing-based methods (e.g. Geo-seq and tomo-seq) rely on microdissection for transcriptome-wide data acquisition from fresh-frozen or FFPE samples [9]. *In situ* sequencing-based methods (e.g. STARmap, STARmap PLUS, Image-seq, and ISS) directly analyze RNA transcripts within cells and tissue sections [9]. *In situ* spatial barcoding-based methods (e.g. 10$\times $ Genomics Visium, Slide-seq, Slide-seqV2, DBiT-seq, XYZeq, sci-Space, Stereo-seq, and Pixel-seq) use spatial barcodes to map RNAs or molecules in fresh-frozen or FFPE samples [9]. From a biological standpoint, sequencing-based SRT technologies are particularly useful for mapping tissue architectures, identifying cell types, and studying their interactions across large tissue sections. These methods have been instrumental in characterizing the spatial distribution of cell types in organs like the brain, kidney, and lung [15]. The biological differences between various sequencing-based SRT methods arise primarily from their spatial resolution, RNA capture efficiency, and how well they maintain the native tissue architecture during sample preparation. For example, 10$\times $ Genomics Visium captures mRNA with spatial probes printed onto slides, offering a center-to-center distance of 100 $\mu m$ and a spot diameter of 55 $\mu m$. Although this is smaller than earlier ST methods, it is still larger than most single cells, leading to transcript contamination from multiple cells [9]. In contrast, Slide-seq and Slide-seqV2 use beads with spatial barcodes laid on flat surfaces, achieving a spatial resolution of 10 $\mu m$, closely approaching single-cell level; high-definition spatial transcriptomics (HDST) further pushes this to 2 $\mu m$ [10]. While Slide-seq and HDST offer superior spatial resolution, they suffer from lower RNA capture efficiencies, limiting their ability to detect enough transcripts for reliable single-cell analyses [9]. Technical challenges specific to *in situ* spatial barcoding-based methods include constructing effective “capturing areas” to deliver barcodes, managing the number of barcode probes per spot (affecting capture efficiency), and balancing spatial resolution (determined by spot size and distance) against capture area and throughput [9]. Different SRT platforms yield datasets with varying biological insights. Technologies like Stereo-seq exhibit the highest capturing capabilities and provide dense transcriptomic data, enhancing the detection of tissue structures and facilitating downstream tasks such as clustering, region annotation, and cell–cell communication analysis [8]. Stereo-seq also generates more sequencing reads for the same tissue region compared with other platforms like Slide-seqV2 or DBiT-seq [8]. Probe-based approaches such as Visium show improved total Unique Molecular Identifier (UMI) counts, possibly due to better read-capturing efficiency or over-quantification [8]. The diversity among SRT technologies is substantial. 10$\times $ Genomics Visium achieves a spatial resolution of 100 $\mu m$ center-to-center with a spot diameter of 55 $\mu m$, capturing $\sim $15 377 UMIs per 55$\times $55 $\mu m$ area in the mouse olfactory bulb, offering multi-cell capture with relatively high UMI counts. Slide-seq achieves a 10 $\mu m$ resolution but with much lower capture efficiency, recording around 59 UMIs per 10$\times $10 $\mu m$ in E12.5 mouse embryos, although it nearly reaches single-cell resolution. Slide-seqV2 maintains the 10 $\mu m$ resolution but improves capture efficiency significantly, with 550 UMIs per 10$\times $10 $\mu m$ area in E12.5 mouse embryos [9]. HDST achieves even finer resolution at 2 $\mu m$, approaching subcellular resolution but with low capture efficiency [9]. XYZeq operates at a coarser 500 $\mu m$ spatial resolution but enables single-cell capture with around 1009 UMIs and 456 genes detected per cell in mouse liver and tumor tissues [9]. sci-Space offers a spatial resolution of 222 $\mu m$ and captures $\sim $2514 UMIs and 1231 genes per cell in E14.0 mouse embryos [9]. Pixel-seq improves spatial granularity to about 1 $\mu m$ resolution, yielding around 977 UMIs per 10$\times $10 $\mu m$ area in the mouse olfactory bulb [9]. Seq-Scope achieves an even finer 0.6 $\mu m$ resolution and captures around 1000 UMIs per 10$\times $10 $\mu m$ area in mouse liver tissues [9]. Although technologies like Stereo-seq, Slide-seq, Slide-seqV2, and HDST push spatial resolution to subcellular levels, challenges remain in achieving high RNA capture efficiencies, which are crucial for accurate single-cell analyses [9]. Overall, spatial transcriptomics is a rapidly evolving field with ongoing challenges. Although significant strides have been made, including the emergence of ultra-high-resolution methods and improved capture techniques, no single method excels across all metrics, such as spatial resolution, transcriptome coverage, and RNA capture efficiency [8, 9]. Method selection depends heavily on the biological question, tissue type, and required resolution [8]. Furthermore, the absence of comprehensive benchmarking studies for sequencing-based SRT platforms complicates method comparisons [8]. Although SRT has benefits, it still deals with issues such as technical biases (batch effects), inconsistent preprocessing methods, and data sparsity (dropouts where expressed genes go undetected). Various computational pipelines and algorithms have been proposed over the years, such as various data imputation techniques, to solve these issues. The computational pipeline for analyzing spatial transcriptomics data involves several intricate steps: generating the spatial matrix by decoding barcodes, image registration, and cell segmentation, preprocessing, clustering, spatial domain identification, cell type annotation, and cell communication inference [9]. A major computational hurdle lies in data sparsity, especially in high-resolution SRT datasets that suffer from low RNA capture per spot or bead, resulting in sparse expression matrices that hinder robust downstream analyses [9]. To address sparsity, gene expression imputation becomes critical. Current approaches leverage spatial smoothing, neighboring spot information, or graph-based inference to recover missing expression values, enhancing the biological interpretability of the data [9]. However, achieving accurate imputation without introducing artifacts remains challenging, especially in regions with genuine biological heterogeneity. Researchers have proposed various imputation methods over the years. These include methods applied to general high-dimensional tabular data, specialized in scRNA-seq or SRT-based data. State-of-the-art (SOTA) methods in general tabular data include K-nearest neighbors (KNN) Impute, SoftImpute, SimpleImpute, etc [16–18]. Conversely, there exist numerous imputation methods on scRNA-seq [19–23]. Since SRT technologies are newer and reaching single-cell resolution, there exist only a few imputation methods on SRT datasets. Hence, Standard imputation methods are yet to be developed for gene-sequencing-based SRT methods. Imputation methods in SRT datasets can be either reference-free (only uses spatial gene expression) or reference-based (needs scRNA-seq expression along with spatial gene expression) [24–30]. Due to the limited number of imputation methods developed on SRT datasets, especially on the recent technologies like Stereo-seq, Slide-seqV2, XYZeq, sci-Space, evaluation of the different SOTA imputation methods is necessary. Han *et al*. [31] benchmarked scRNA-seq-based imputation methods on scHi-C, whereas Liu *et al*. [32] explored the effectiveness of scRNA-seq-based imputation methods in scATAC-seq data. However, to our knowledge, there are no benchmarking evaluations of imputation methods on recent SRT technologies. Conversely, imputation methods’ performance on SRT datasets usually involves 10$\times $ Genomics Visium and lacks the inclusion of newer technology. Furthermore, the evaluation of these recent methods does not include important metrics such as ARI, NMI, etc., which are important from a biological perspective [26, 27].

Considering the research gap related to the ability of imputation methods on SRT technologies, we first explore the imputation effectiveness of several methods on SRT technologies. In this work, we propose a pipeline to explore the effectiveness of different imputation methods, including those developed for scRNA-seq data, SRT data, and traditional methods on new SRT technology. We measure the effectiveness using clustering accuracy and other relevant metrics. Imputation methods in SRTs are limited and still evolving. Hence, we select the most SOTA imputation methods from the literature based on statistical approaches and deep-learning techniques. Consequently, we select different SRT technologies with ground truth and apply the imputation methods to measure their effectiveness. Furthermore, considering the importance of spatial information of SRT datasets in imputation, we propose a simple and scalable imputation method, SpaMean-Impute, that has the ability to determine the actual dropouts and imputes the necessary dropout locations with the aggregated spot-gene expression value. Our proposed method outperforms the benchmarked imputation methods consistently in different SRT technology datasets, which shows that consideration of spatial information is a must while imputing such datasets.

Hence, the main contributions of this work can be summarized as follows: (i) as previous research lacks any formal evaluation of imputation methods on diverse SRT datasets, we evaluate their effectiveness using a benchmark pipeline and discuss our findings. The benchmarking focuses on understanding whether any imputation method performs consistently well across different SRT technologies, whether certain methods are better suited for specific technologies, and how the selection of top genes affects the performance, (ii) we propose a new algorithm for SRT datasets after evaluating the performance of the previous methods. The proposed method integrates spatial information of the SRT datasets and consistently performs better than these SOTA benchmarked methods across different settings.

## Materials and methods

In this section, we first discuss the benchmarking pipeline and the associated datasets of the SRT technology, dataset preprocessing, imputation methods, and evaluation metrics from Section “Pipeline of the imputation benchmark on SRT Datasets” to “Metrics to evaluate the imputation methods.” Next, we discuss the proposed method, “SpaMean-Impute” in Section “Proposed imputation Framework-SpaMean-Impute.” We demonstrate how our method has the ability to detect valid dropouts utilizing the spatial information of SRT datasets.

### Pipeline of the imputation benchmark on spatially resolved transcriptomics datasets

**Figure 1:**
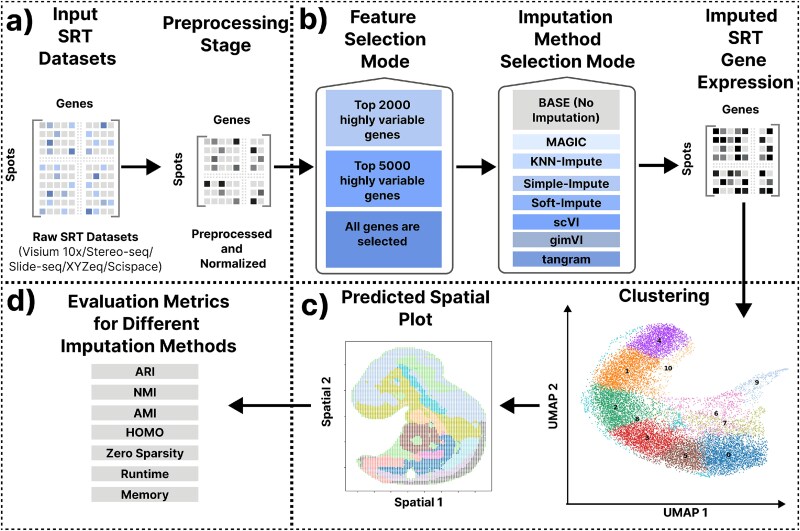
Overview of the computational pipeline for evaluating imputation methods in SRT data. The overall method is divided into four distinct parts: (a) Raw SRT datasets acquisition from multiple platforms and a preprocessing stage involving quality filtering and normalization to ensure consistency and minimize technical noise. (b) Feature selection mode is applied to retain either the top 2000 or 5000 most highly variable genes, or to include all genes. Subsequently, an imputation method is selected from a panel of techniques, including BASE (no imputation), Markov affinity-based graph imputation of cells (MAGIC), KNN-Impute, Simple-Impute, Soft-Impute, scVI, gimVI, and tangram. (c) The imputed data are used to perform dimensionality reduction via UMAP and clustering analysis that enables visualization of cellular and tissue architecture in both low-dimensional embeddings and spatial coordinate plots. The predicted spatial plot provides spatial correspondence of cluster assignments back to the tissue sections. (d) To quantitatively benchmark the effectiveness of each imputation method, multiple evaluation metrics are employed, such as ARI, NMI, AMI, and HOMO, as well as computational and data-quality metrics such as zero sparsity (proportion of zero entries), runtime, and memory consumption. Together, these components constitute a robust framework for the systematic comparison of imputation approaches in SRT analysis.

We present a unified computational pipeline to evaluate the imputation techniques (Section “Imputation methods included in the pipeline”) in SRT datasets. The workflow demonstrated in Fig. 1, begins with the acquisition of raw SRT datasets from platforms such as 10$\times $ Genomics Visium, Stereo-seq, Slide-seqV2, XYZeq, and sci-Space. These datasets are structured as spatial gene expression matrices with spatial spots as rows and genes as columns. The raw datasets undergo a preprocessing stage that is discussed in detail in Section “Dataset preprocessing.”

Following preprocessing, the feature selection stage using Scanpy’s highly variable genes allows the retention of either the top 2000 or 5000 highly variable genes, or the use of all available genes [33]. The selection of genes, i.e. features, is kept in our benchmarking pipeline to observe how the number of features affects the imputation characteristics and downstream analysis. However, the choice of 2000 or 5000 features is due to biological convention in selecting highly variable features. Once gene selection is finalized, an imputation method is applied to reconstruct the missing or sparse gene expression values. Available methods include BASE that performs no imputation, and a range of SOTA imputation techniques (discussed in Section “Imputation methods included in the pipeline”). The pipeline is designed in such a way that additional imputation methods can be easily integrated into our pipeline. The imputed gene expression matrix is then subjected to dimensionality reduction using uniform manifold approximation and projection (UMAP), followed by clustering to identify spatially coherent regions [34]. The resulting clusters are visualized both in the reduced UMAP space and in their original spatial coordinates to interpret the spatial context of biological structures and patterns.

Finally, the performance of each imputation method is assessed using the evaluation metrics mentioned in section “Metrics to evaluate the imputation methods.” Additionally, zero sparsity is used to quantify the reduction of zero entries post-imputation, while runtime and memory usage are monitored to evaluate the computational efficiency and resource demands of each method. This comprehensive methodology provides a robust framework for benchmarking imputation strategies in SRT data analysis. The detailed results of this benchmarking pipeline across all the SRT datasets are discussed in the Results section.

### Datasets

The study employed datasets from five SRT technologies: *10$\times $ Genomics Visium, Stereo-seq, Slide-seqV2, XYZeq, and sci-Space* to benchmark imputation. The *DLPFC* and *BRCA* dataset was produced using the *10$\times $ Genomics Visium* platform, which captures transcriptome-wide expression with a spatial resolution of $\sim $*55$\mu $m* [35, 36]. *DLPFC* dataset profiled the human dorsolateral prefrontal cortex from 12 sections across neurotypical adult donors and provided high-quality data for identifying laminar-specific gene expression, making it a common benchmark for spatial clustering methods [35]. Conversely, the *BRCA* dataset mapped gene expression across human breast cancer tissue over *3798 spots and 36601 genes* [36]. *Stereo-seq (Spatiotemporal Enhanced Resolution Omics-sequencing)* is a high-resolution spatial transcriptomics technology that captures genome-wide gene expression with spatial fidelity using DNA nanoball (DNB)-patterned arrays [37]. The datasets we utilized for Stereo-seq contained bin having 50$\times $50 DNBs spaced 715 nm apart, providing a resolution of 36$\times $36 $\mu $m per bin [37]. For the mouse liver atlas, 878 334 non-overlapping bins covering 11.22 cm$^{2}$ were profiled and integrated with scRNA-seq data from 473 290 cells (435 413 newly generated), resulting in the identification of 8127 zonated genes and 1140 ligand–receptor interactions (686 zonated), thereby enabling construction of a high-definition spatial–temporal atlas of liver homeostasis and regeneration [37]. The *Slide-seqV2* dataset utilized a bead-based barcoding strategy that achieves *10$\mu $m resolution*, enabling near-cellular transcriptome profiling with markedly higher sensitivity than its predecessor [38]. It has been used to explore spatial gene expression in mouse brain tissues, including the hippocampus and embryonic neocortex, recovering a high number of UMIs per feature and allowing integration with trajectory analysis tools [38]. *sci-Space (Spatial Combinatorial Indexing Sequencing)* is another method enabling single-cell resolution across large tissue areas [39]. It integrates combinatorial indexing with spatially patterned oligo hashing on glass slides. Srivatsan *et al*. [39] profiled 14 sagittal sections from E14.0 mouse embryos, generating 121 909 spatially resolved single-cell transcriptomes (mean 2514 UMIs and 1231 genes per cell), covering 15 102 spatial positions (8.1 nuclei/position) over an 18$\times $18 mm area with 7056 unique hashing spots at $\sim $200 $\mu $m resolution. Spatial localization was validated through fluorescent waypoints, and data were integrated with existing atlases. The approach allows cell-type-specific spatial analysis, detection of spatially patterned gene expression (e.g. *Slc6a3* and *Cyp26b1*), and reveals spatially informed pseudotemporal trajectories, enabling high-resolution spatial atlases of development. Furthermore, *XYZeq* dataset is also integrated in our benchmarking, where this dataset leveraged a two-round split-pool indexing strategy to encode spatial metadata directly into scRNA-seq libraries, achieving spatial resolution of $\sim $**500 $\mu $m** [40]. Applied to murine tumor models, XYZeq enabled spatially resolved transcriptome profiling at single-cell resolution. Together, these datasets offer complementary resolution scales and tissue types, providing a robust foundation for benchmarking and evaluating the performance of computational methods. The details of the dataset’s size are summarized in Table 1. We selected these datasets because they have proper annotations that are necessary to evaluate the imputation capability through downstream analyses.

**Table 1:** SRT datasets for five technologies used in our benchmarking

SL No.	Technology	Dataset name	Dataset size before preprocessing (Spots $\times $ Genes)	Dataset size after preprocessing (Spots $\times $ Genes)
01.	10$\times $ Genomics Visium [35, 36]	151507	4221 $\times $ 33 538	2890$\times $15 274
		151508	4381 $\times $ 33 538	2549 $\times $ 14 507
		151509	4788 $\times $ 33 538	4184 $\times $ 15 892
		151510	4595 $\times $ 33 538	3722 $\times $ 15 111
		151669	3636 $\times $ 33 538	1333 $\times $ 14 539
		151670	3484 $\times $ 33 538	1102 $\times $ 13 923
		151671	4093 $\times $ 33 538	1808 $\times $ 14 976
		151672	3888 $\times $ 33 538	1848 $\times $ 14 816
		151673	4221 $\times $ 33 538	2890 $\times $ 15 274
		151674	3635 $\times $ 33 538	3197 $\times $ 7130
		151675	3566 $\times $ 33 538	2081 $\times $ 15 148
		151676	3431 $\times $ 33 538	2220 $\times $ 15 434
		BRCA1	3798 $\times $ 36 601	3169 $\times $ 18 968
02.	Stereo-seq [37]	DT2_D0	42 741 $\times $ 29 075	23 259 $\times $ 16 865
		DX6_D2	14 929 $\times $ 29 075	14 455 $\times $ 17 864
		FB2_D1	16 264 $\times $ 29 075	16 211 $\times $ 18 252
03.	Slide-seqV2 [38]	WT	31 659 $\times $ 24 450	9008 $\times $ 16 598
		diabetes	27 194 $\times $ 24 420	9435 $\times $ 16 884
		mouse	41 786 $\times $ 4000	22 560 $\times $ 3919
04.	sci-Space [39]	GSE166692	122 278 $\times $ 52 636	9517 $\times $ 24879
05.	XYZeq [40]	GSE164430	7505 $\times $ 52 025	2914 $\times $ 25 556
		GSM5009529	6447 $\times $ 121 472	4293 $\times $ 19 390
		GSM5009539	2703 $\times $ 9776	2501 $\times $ 8776

Dataset size indicates (Spots $\times $ Genes), and the table demonstrates both the size before preprocessing (i.e. Raw), and the size after preprocessing.

We can observe in Table 1 that 13 datasets from the 10$\times $ Genomics Visium platform are used, with spot counts ranging from 1102 to 4184 and gene counts between 7130 and 18 968. Stereo-seq datasets include DT2_D0, DX6_D2, and FB2_D1, with spot counts ranging from 14 455 to 23 259. Slide-seqV2 comprises three datasets—WT, diabetes, and mouse—featuring spot counts between 9008 and 22 560. For XYZeq, three datasets were used, including GSE164430, with a maximum gene count of 25 556. Lastly, sci-Space includes one dataset (GSE 166692) containing 9517 spots and 24 879 genes. We can observe from the dataset size that Stereo-seq and Slide-seqV2 have the maximum number of spots detected in tissues, whereas 10$\times $ Genomics Visium and XYZeq have the lowest. These datasets have been widely regarded as “gold-standard” references within the field and are commonly used in benchmarking studies. Crucially, these datasets provide ground truth annotations and comprehensive metadata, which are essential for rigorously evaluating the performance of imputation methods on downstream analyses such as clustering and cell-type assignment. Using these well-characterized datasets allowed us to compare methods under consistent and transparent conditions. We also carefully reviewed recent developments in SRT technologies and datasets. Despite substantial advances in spatial resolution and gene-capture chemistry, low probe/molecule capture efficiency, limited sequencing depth, and RNA degradation remain significant challenges, as evident from the recent literature [41]. This is evident across multiple platforms. For example, the widely used Visium platform continues to show systematic gene-specific biases in a recent study, missing key marker genes, such as Cdc42 and Cx3cl1, despite their inclusion in the probe set [8, 42]. Likewise, even highly sensitive imaging-based methods detect fewer transcripts overall [43, 44]. In addition, current sequencing runs across multiple platforms still fail to reach saturation, indicating that under-sequencing remains a pervasive issue [8]. These lower library sizes can make it difficult to detect important genes, particularly those that are lowly expressed [41]. We specifically examined Stereo-seq V2 that represents one of the most advanced technologies currently available. Stereo-seq V2 uses random primers that improve capture efficiency over traditional poly(T) primers and perform better on fragmented RNA from FFPE samples [44]. However, even Stereo-seq V2 experiences inherent limitations: because of its relatively short read length and broad genome coverage, reads covering specific regions remain low [42]. High dropout rates and sparse count matrices are still described as “inherent” to SRT data and present computational challenges such as algorithm instability [45, 46]. Finally, while data quality has improved in terms of spatial resolution and sensitivity, it remains variable and influenced by factors such as molecular diffusion, noise, and artifacts. Molecular diffusion during sample preparation can blur the spatial location of transcripts, reducing the effective resolution of the technology [43]. SRT data are also susceptible to various sources of noise, including ambient RNA, signal spillover between spots, and transcripts from incomplete cells [45], and sample preparation can introduce artifacts such as blood contamination [8]. In addition, trade-offs between sensitivity and transcript detection persist across platforms [8]. This diverse selection ensures broad coverage across widely used SRT technologies.

### Dataset preprocessing

All SRT datasets underwent a standardized quality control (QC) and preprocessing workflow, with slight adjustments based on dataset-specific characteristics, such as size and quality. The preprocessing was performed using the scanpy library [33].

First, mitochondrial genes were annotated by identifying gene names starting with “MT-.” QC metrics per cell were calculated, including the total counts of UMI, the number of genes detected, and the percentage of counts attributed to mitochondrial genes. Spots with extremely low or high total counts (likely representing empty or doublet-containing barcodes) were filtered. The filtering thresholds varied across datasets: the min_counts parameter ranged from 50 to 500, depending on the dataset’s overall quality and sequencing depth, while max_counts was typically set around 35 000. Cells with >20% mitochondrial gene expression were excluded to remove potentially unhealthy or dying cells. Next, genes detected in fewer than a threshold number of spots were removed to reduce noise; the min_cells threshold was set between 10 and 50, depending on the dataset. Following filtering, the datasets were normalized using total-count normalization, and log-transformed after adding a pseudocount of 1. These preprocessing steps ensured that downstream analyses were performed on high-quality, comparable gene expression matrices across technologies. Finally, the preprocessed dataset from all raw versions of the datasets is saved in ’.h5ad’ AnnData format to facilitate easier handling in subsequent benchmarking analyses. The size of the preprocessed datasets after preprocessing can be observed in Table 1 with respect to the size of the dataset before preprocessing (i.e. raw versions). All preprocessed datasets can be found in the “Data availability Sections” and in the GitHub link.

### Imputation methods included in the pipeline

We applied seven imputation methods: MAGIC, KNN Impute, SoftImpute, SimpleImpute, scVI, gimVI, and tangram in our study. Each method leverages distinct statistical, machine learning, or deep learning principles to recover missing gene expression signals and improve downstream analysis. These methods were chosen based on their strong performance in previous benchmarks, their broad adoption in the single-cell research community, and their ability to compute tabular data. These methods consist of generalized imputation characteristics (KNN Impute and SimpleImpute), specially designed for scRNA-seq datasets (such as MAGIC, SoftImpute, and scVI) or designed SRT datasets (gimVI and tangram). Such diverse methods are selected to assess their capability in imputing dropouts in SRT datasets.

MAGIC is a diffusion-based imputation method that constructs a graph where nodes represent cells, and edges reflect cell-to-cell similarity based on gene expression profiles [47]. The algorithm applies a Markov diffusion operator to spread gene expression information across the graph, effectively smoothing expression values, and recovering gene–gene relationships lost due to dropout events. This method is particularly useful for visualizing continuous trajectories in cell differentiation processes.

KNN impute relies on the principle that similar cells exhibit similar gene expression patterns. For each cell with missing values, the algorithm identifies the KNN based on Euclidean distance in gene expression space [16, 18]. Missing entries are imputed by averaging the corresponding values across these neighbors. While simple, this method provides a non-parametric and intuitive baseline for imputing sparse gene matrices, especially in datasets with a clear clustering structure. However, this method may fail in sparse tabular data due to sparsity among the KNN.

SoftImpute implements a matrix completion strategy via low-rank approximation. Specifically, it iteratively performs soft-threshold singular value decomposition to minimize the reconstruction error over observed entries while shrinking singular values to enforce a low-rank structure [17]. This approach assumes the gene expression matrix is approximately low rank and is well-suited for datasets with random missingness and low technical noise. However, this approach assumes ‘NaN’ value for the dropouts, i.e. requiring prior knowledge of the dropouts. We converted all the zero values in the SRT datasets to ’NaN’ and then applied SoftImpute.

SimpleImpute from scikit-learn is also employed as a benchmark imputation method due to its simplistic nature in imputing tabular data. It fills missing values using basic statistical measures such as the mean, median, or most frequent value calculated independently for each gene across all cells [18]. This univariate approach does not incorporate any structure or dependencies within the data, such as gene–gene correlations or cell–cell similarities. Despite its simplicity, it provides a useful baseline for assessing the relative performance of more sophisticated imputation models. SimpleImpute similarly requires prior knowledge of the dropouts and replacement of the dropouts with ‘NaN’.

Single-cell variational inference (scVI) is a deep generative model based on a variational autoencoder (VAE) framework [48]. It models gene expression counts as samples from a negative binomial distribution, parameterized by latent variables inferred from the data. scVI accounts for batch effects and overdispersion and captures complex, nonlinear gene dependencies through its learned latent space. This probabilistic framework allows for robust denoising, imputation, and latent representation of high-dimensional scRNA-seq data.

Conversely, gimVI extends scVI to jointly model paired or unpaired single-cell and SRT data [24]. It incorporates a multi-view VAE structure where one encoder processes the spatial modality and another encoder handles the single-cell modality. A shared latent space is learned, enabling the model to predict unmeasured genes in spatial data by transferring information from the well-characterized single-cell reference.

Another mapping framework is *tangram* that aligns scRNA-seq profiles to SRT coordinates by solving an optimization problem that minimizes the difference between the observed spatial gene expression and a weighted sum of single-cell profiles [25]. It operates under the assumption that spatial tissue sections can be explained as mixtures of cell types or states observed in scRNA-seq data. ’tangram’ produces a probabilistic assignment matrix, enabling high-resolution spatial reconstruction of cellular composition across tissue sections.

These diverse imputation methods are used to impute the five SRT technologies’ 23 datasets and subsequently evaluate these methods’ imputation capability.

### Metrics to evaluate the imputation methods

To evaluate the performance of imputation methods on SRT datasets, we employ a set of well-established clustering evaluation metrics. These metrics compare predicted clusters, $ P $, (e.g. using the Leiden method) obtained from imputed gene expression profiles against known biological annotations, i.e. true spatial domain labels or manually annotated layers, $ T $.

Adjusted rand index (ARI): ARI quantifies the agreement between predicted clustering assignments, $ P $ and true biological labels, $ T $ [49]. It corrects for chance grouping by comparing the number of cell/spot pairs that are consistently grouped or separated in both partitions. ARI ranges from −1 (no agreement) to 1 (perfect agreement), with 0 representing chance-level agreement. The equation of ARI is given as follows [50]:


(1) \begin{align*}& ARI = \frac{ \sum_{ij} \binom{n_{ij}}{2} - [ \sum_{i} \binom{a_{i}}{2} \sum_{j} \binom{b_{j}}{2}] / \binom{n}{2} } { \frac{1}{2} \left[ \sum_{i} \binom{a_{i}}{2} + \sum_{j} \binom{b_{j}}{2} \right] - [ \sum_{i} \binom{a_{i}}{2} \sum_{j} \binom{b_{j}}{2}] / \binom{n}{2} }\end{align*}

Here, $ n_{ij} $ denotes the number of spots belonging to both $ i $th true class and $ j $th predicted cluster. This value reflects how many matched pairs exist between the true and predicted labels. Similarly, $ a_{i} $ represents the number of samples in the $ i $th true class, and $ b_{j} $ represents the number of samples in the $ j $th predicted labels. The total number of samples is denoted by $ n $. The corresponding pairs among true and predicted labels are computed for expected agreement by random assignments. By calculating the actual agreement between true pairs and predicted pairs between spots over the random assignments, the numerator of ARI measures how much better the clustering is than random chance. The denominator captures the maximum number of pairwise agreements possible, given the distributions of true and predicted labels. By normalizing in this way, ARI provides a chance-corrected measure of clustering accuracy, where 1 indicates perfect agreement, and 0 indicates agreement no better than random.

**Normalized mutual information (NMI)**: NMI measures the mutual dependence between predicted clusters and ground truth labels. It is based on the mutual information (MI), which quantifies how much knowing the predicted label reduces the uncertainty of the true label [51]. NMI scales MI between 0 and 1, making scores comparable across datasets. The equation of NMI is as follows:


(2) \begin{align*} \mathrm{MI}(T, P) &= H(T) - H(T|P) \end{align*}

(3) \begin{align*} \mathrm{NMI} &= \frac{\mathrm{MI}(T, P)}{\sqrt{H(T) \cdot H(P)}} \end{align*}

Where $ T $ and $ P $ are the true and predicted label sets, respectively, and $ H(\cdot ) $ denotes Shannon entropy. $ H(T) $ is the entropy of the true labels and $ H(T|P) $ is the conditional entropy of true labels given predicted clusters. Their difference, the mutual information $ \mathrm{MI}(T, P) $, quantifies the reduction in uncertainty about $ T $ when $ P $ is known [51].

**Adjusted mutual information (AMI)**: AMI improves upon NMI by correcting for the expected MI under random assignments [51]. This makes it especially robust for unbalanced or skewed class distributions, which are common in real SRT datasets. The equation for AMI is as follows:


(4) \begin{align*} \mathrm{AMI} &= \frac{\mathrm{MI}(T, P) - \mathbb{E}[\mathrm{MI}(T, P)]}{\mathrm{avg}(H(T), H(P)) - \mathbb{E}[\mathrm{MI}(T, P)]}\end{align*}

Here, $ \mathbb{E}[\mathrm{MI}(T, P)] $ is the expected mutual information between $ T $ and $ P $ under a random model.

**Homogeneity (HOMO)**: HOMO assesses whether each predicted cluster contains only spots from a single ground truth label. A perfectly homogeneous clustering (score = 1) means that no cluster mixes spots from different biological classes. The equation for HOMO is shown below [52]:


(5) \begin{align*} \mathrm{Homo} &= 1 - \frac{H(T|P)}{H(T)}\end{align*}

Where $ H(T|P) $ is the conditional entropy of predicted labels $ T $ given the true labels $ P $. Lower conditional entropy (i.e. higher HOMO) implies that each predicted cluster is dominated by a single ground truth class.

Among these metrics, AMI is theoretically preferred over NMI, although both typically yield similar results. Compared with ARI, AMI is more suitable when cluster sizes are unbalanced or include small clusters [51]. Since SRT data often contain rare cell populations, AMI is generally more appropriate, whereas ARI is preferable when cluster sizes are nearly uniform [52]. Together, these metrics provide a comprehensive view of clustering quality, including label agreement, information preservation, and structural integrity of the spatial data after imputation.

### Proposed imputation Framework-SpaMean-Impute

**Figure 2:**
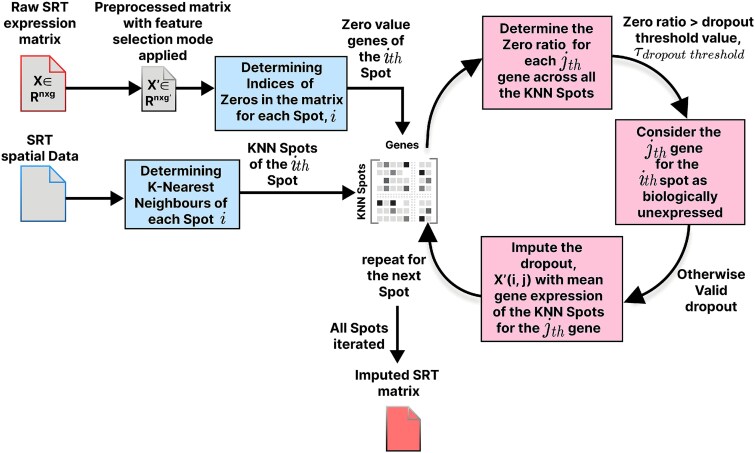
This diagram outlines the steps used in SpaMean-Impute to impute zero gene expression values in SRTs. Starting with a filtered expression matrix and spatial coordinates, the method identifies zero entries per spot and locates the $k$-nearest neighbors, where for each zero, a submatrix of neighbor values is extracted, and the zero ratio is computed and if the ratio is below a set threshold, the value is imputed using the average of non-zero neighbor values, this approach recovers likely technical dropouts while preserving true biological sparsity.

In this work, we also propose a simple imputation framework, “SpaMean-Impute,” utilizing the spatial information in the datasets. The overall method can be divided into four parts: (i) Finding the valid dropouts and true biological zeros, (ii) similar spots determination of each spot using spatial coordinates, (iii) zero ratio determination across similar spots for differentiating between dropouts and biological zeros, and finally, (iv) imputing the valid dropouts. The proposed methodology is depicted in Fig. 2.

#### Finding the indices of zeros in the spot–gene matrix

Given a gene expression matrix $\mathbf{X} \in \mathbb{R}^{n \times g}$, where each row represents a spatial location (or spot) and each column represents a gene. This expression matrix is processed after applying the top genes selection mode, we get another matrix, $\mathbf{X}^{\prime} \in \mathbb{R}^{n \times g^{\prime}}$ ($g^{\prime}$ is the number of genes selected for the final imputation stage). Now, the goal is to identify all zero-valued entries. These zeros often correspond to dropouts and are targeted for potential imputation. We define a mapping from each spot $i \in \{1, 2, \dots , n\}$ to the set of gene indices $j \in \{1, 2, \dots , g^{\prime}\}$ for which the expression value $X^{\prime}_{ij} = 0$. Let $\mathbb{1}_{\{X^{\prime}_{ij} = 0\}}$ be the indicator function that returns 1 if $X^{\prime}_{ij} = 0$ and 0 otherwise. Then, for each spot $i$, the set of zero-valued gene indices is defined as:


\begin{align*} & \mathcal{Z}_{i} = \left\{ j \in \{1, 2, \dots, g\} \mid X^{\prime}_{ij} = 0 \right\} \end{align*}

These sets $\mathcal{Z}_{i}$ are stored in a dictionary-like structure:


\begin{align*} & \mathrm{zero}{\_}\mathrm{dict}{\_}\mathrm{spot}[i] = \mathcal{Z}_{i} \end{align*}

Additionally, to reduce computational complexity, any gene $j$ with zero expression across all spots is first removed to ensure that only informative genes are retained:


\begin{align*} & \mathbf{X}^{\prime} = \mathbf{X}^{\prime}\left[:,\ j \text{ such that}\ \sum_{i=1}^{n} \mathbb{1}_{\{X^{\prime}_{ij} \neq 0\}}> 0\right] \end{align*}

#### Determining the nearest spots using spatial coordinates

Each spot in SRT is associated with a 2D spatial coordinate representing its physical location on the tissue slide. Let the coordinate matrix be denoted by:


\begin{align*} & \mathbf{S} \in \mathbb{R}^{n \times 2}, \quad \mathrm{where} \quad \mathbf{S}_{i} = (x_{i}, y_{i}) \end{align*}

for spot $i = 1, 2, \dots , n$. To perform local neighborhood-based imputation, we compute the $k$-nearest spatial neighbors for each spot using Euclidean distance. The Euclidean distance $d_{ij}$ between any two spots $i$ and $j$ is given by:


\begin{align*} & d_{ij} = \lVert \mathbf{S}_{i} - \mathbf{S}_{j} \rVert_{2} = \sqrt{(x_{i} - x_{j})^{2} + (y_{i} - y_{j})^{2}} \end{align*}

For each spot $i$, we find a set of $k$-nearest neighbors (excluding the spot itself), denoted by:


\begin{align*} & \mathcal{N}_{i}^{(k)} = \operatorname{argsort}_{j \ne i}(d_{ij})[:k] \end{align*}

This results in a neighbor dictionary:


\begin{align*} & \mathrm{neighbors}{\_}\mathrm{dict}[i] = \mathcal{N}_{i}^{(k)} \end{align*}

This spatially derived neighbor list serves as the foundation for imputing gene expression values from nearby spots, leveraging the spatial smoothness inherent in tissue structure.

#### Submatrix extraction and zero-ratio calculation across K-nearest neighbors spots

After determining the $k$-nearest neighbors for each spot $i$, denoted as $\mathcal{N}_{i}^{(k)}$, and identifying the indices of genes that have zero expression in spot $i$, denoted as:


\begin{align*} & \mathcal{Z}_{i} = \left\{ j \mid X^{\prime}_{ij} = 0 \right\} \end{align*}

A submatrix $\mathbf{X^{\prime}}_{i}^{\mathrm{sub}} \in \mathbb{R}^{k \times |\mathcal{Z}_{i}|}$ is extracted that contains the expression values of those genes across the $k$ neighbors of spot $i$:


\begin{align*} & \mathbf{X^{\prime}}_{i}^{\mathrm{sub}} = \left[ X^{\prime}_{nj} \right] \quad \mathrm{for} \quad n \in \mathcal{N}_{i}^{(k)}, \; j \in \mathcal{Z}_{i} \end{align*}

To assess the reliability of imputing a zero entry, we compute the zero ratio for each gene $j$ across its neighborhood. This is the fraction of neighbor spots where gene $j$ is also zero:


\begin{align*} & \mathrm{zero}{\_}\mathrm{ratio}_{ij} = \frac{ \sum\limits_{n \in \mathcal{N}_{i}^{(k)}} \mathbb{1}_{X^{\prime}_{nj} = 0}} {k} \end{align*}

This ratio helps in deciding whether the gene $j$ in spot $i$ should be imputed. Only if $\mathrm{zero}{\_}\mathrm{ratio}_{ij} \leq \delta $, where $\delta $ is the dropout threshold (e.g. $\delta = 0.4$), the imputation is allowed. This is from the biological perspective that higher zero ratios may indicate true zero expression where lower zero ratios in a gene across KNN spots may indicate a technical error in the data and need imputations. Hence, the set of valid genes to be imputed for spot $i$ is:


\begin{align*} & \mathcal{Z}_{i}^{(\mathrm{valid})} = \left\{ j \in \mathcal{Z}_{i} \; \middle| \; \mathrm{zero}{\_}\mathrm{ratio}_{ij} \leq \delta \right\} \end{align*}

#### Mean-based imputation using K-nearest neighbors

Once the set of valid zero-expression genes for each spot $i$ has been filtered using the zero-ratio criterion (i.e. $\mathcal{Z}_{i}^{(\mathrm{valid})}$), the next step is to impute those missing values using the average expression values from the neighboring spots. $\mathcal{N}_{i}^{(k)}$ denote the $k$-nearest neighbors of spot $i$ and $\mathbf{X}^{\prime} \in \mathbb{R}^{n \times g^{\prime}}$ is the gene expression matrix. For each gene $j \in \mathcal{Z}_{i}^{(\mathrm{valid})}$ of spot $i$, the imputed expression value is computed as the mean of non-zero expression values of gene $j$ across the neighbors of spot $i$:


\begin{align*} & X_{ij}^{(\mathrm{imputed})^{\prime}} = \frac{ \sum\limits_{m \in \mathcal{N}_{i}^{(k)} \; \wedge \; X^{\prime}_{mj} \ne 0} X^{\prime}_{nj}} { |\mathcal{N^{\prime}}_{i}^{(k)}| } \end{align*}

where,


\begin{align*} & \mathcal{N^{\prime}}_{i}^{(k)} = \left\{ \mathcal{N}_{i}^{(k)} \middle| X^{\prime}_{nj}\ne 0 \right\} \end{align*}

If all neighbors have zero values for gene $j$ (i.e. the denominator is zero), then the imputation for that entry is skipped to avoid division by zero:


\begin{align*} & X_{ij}^{(\mathrm{imputed})^{\prime}} = \begin{cases} \text{mean of non-zero} X_{nj} & \mathrm{if}\ \exists \, n \in \mathcal{N}_{i}^{(k)} \text{ such that}\ X_{nj} \ne 0 \\ \text{0 (unchanged)} & \mathrm{otherwise} \end{cases} \end{align*}

After computing the imputed values for all $j \in \mathcal{Z}_{i}^{(\mathrm{valid})}$, they are inserted back into the original gene expression matrix:


\begin{align*} & X_{ij}^{\prime} \leftarrow X_{ij}^{(\mathrm{imputed})^{\prime}}, \quad \forall j \in \mathcal{Z}_{i}^{(\mathrm{valid})} \end{align*}

This approach assumes that the non-zero expression values from neighboring spots can provide reliable estimates for the missing gene expression values at spot $i$, particularly when the surrounding cells show consistent expression for that gene. This process continues until it iterates over all the spots. The pseudo code of our proposed method is shown below (Algorithm 1). This algorithm performs local imputation of missing gene expression values by identifying the indices of genes with zero expression and the $k$-nearest neighboring spots. These neighbors serve as a local reference for estimating missing values. For each zero-valued gene in a spot, the algorithm calculates the proportion of zero values (zero ratio) for that gene across the neighborhood. If the ratio is below a predefined threshold $\delta $, the gene is considered eligible for imputation by averaging the non-zero values of the gene from the neighboring spots. This strategy prevents over-smoothing and preserves biologically meaningful sparsity, as it avoids imputing genes that are likely truly unexpressed in a local context.

## Results and discussion

Performance evaluation of the SOTA imputation and the proposed method is discussed in this section. The benchmarking is performed centered on several research questions (RQs) such as: (i) Is there any imputation method that consistently performs well across all SRT technologies?, (ii) Is there any method that performs well in a particular technology?, and (iii) Do the top gene selections impact the performance of the methods?. The following subsequent sections will discuss the findings based on these RQs, and lastly, the performance of ‘SpaMean-Impute’ compared with the SOTA methods will be discussed.


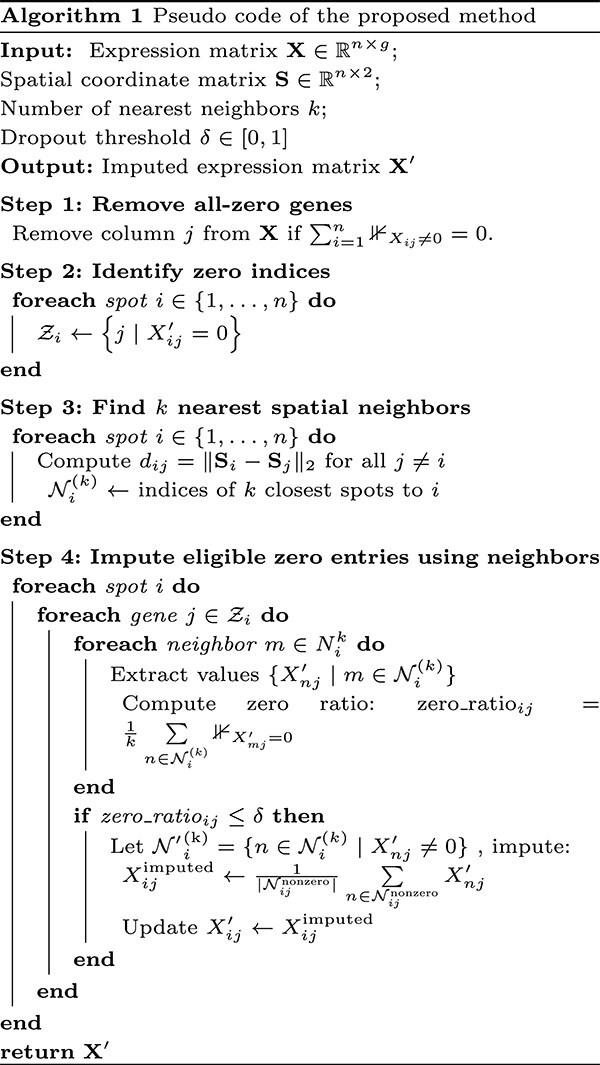


### Evaluation of imputation methods using clustering-based metrics

The benchmarking evaluates imputation methods against the base method (i.e. no imputation) across five technology datasets, using ARI, NMI, AMI, and HOMO as performance metrics. We shall discuss these comparisons with respect to SRT technologies.

#### Performance on 10$\times $ Genomics Visium

**Figure 3:**
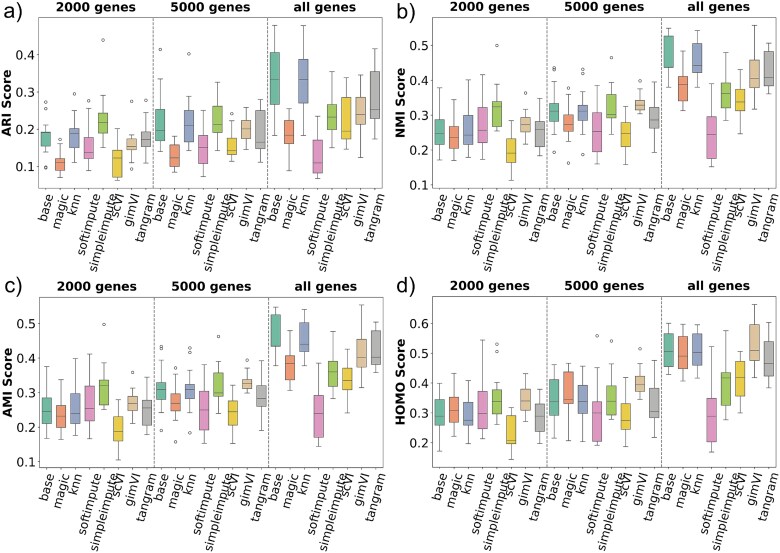
Clustering benchmark results for the 10$\times $ Genomics Visium dataset across three gene selection levels (2000 genes, 5000 genes, and all genes). Eight methods—including the base (no imputation) and seven imputation approaches—are evaluated using (a) ARI, (b) NMI, (c) AMI, and (d) HOMO scores. gimVI performs well in most cases. However, the base method performs well when all genes are selected.

Figure 3 demonstrates imputation methods’ performance for Visium datasets. ARI results reveal that most of the imputation method struggles with clustering accuracy compared with the base method (Fig. 3a). The gene selection also has an impact, where the method performs poorly when the top 2000 or 5000 genes are selected, compared with when all the genes are selected. Both NMI and AMI values highlight the superior performance of KNN Impute gimVI, and tangram when all genes are selected, whereas SimpleImpute performed better when 2000 or 500 genes are selected. Lastly, HOMO scores indicate that gimVI offers the most homogeneous clustering, displaying stable and high values across various gene subsets. Overall, **gimVI** performed well for most cases 10$\times $ Genomics Visium. It is also interesting to notice that the mean ARI, NMI, AMI, and HOMO scores are better for base and across all imputation methods when ’all genes’ are selected for the 10$\times $ Genomics Visium.

#### Performance on stereo-seq

**Figure 4:**
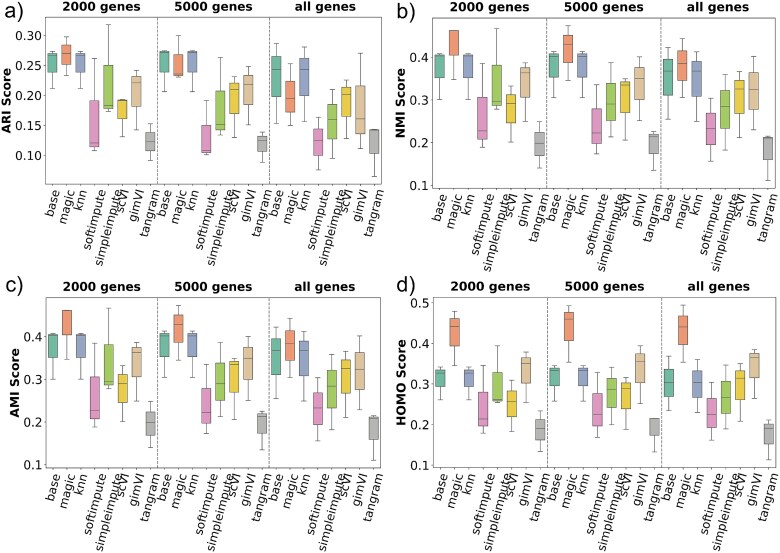
Clustering benchmark results for the Stereo-seq dataset across three gene selection levels (2000 genes, 5000 genes, and all genes). Eight methods—including the base (no imputation) and seven imputation approaches—are evaluated using (a) ARI, (b) NMI, (c) AMI, and (d) HOMO scores. MAGIC method consistently performs well across all metrics

Figure 4 presents comparisons of the methods’ performance for the Stereo-seq technology. The base method consistently achieves strong clustering performance, particularly with ARI, outperforming most imputation methods across all gene subsets except for **MAGIC**. In contrast, more complex methods like scVI, gimVI, and tangram tend to show reduced performance in both clustering accuracy and stability. Although KNN Impute, gimVI provides relatively stable and homogeneous clustering in the HOMO score, it does not consistently outperform the base method. These results suggest that aggressive or deep imputation may distort the biological structure of the Stereo-seq data, and simpler methods may be more suitable. Unlike 10$\times $ Genomics Visium the mean ARI, NMI, AMI, and HOMO scores are better for most of the imputation methods (i.e. MAGIC) when 2000 or 5000 genes are selected for the Stereo-seq. This suggests that imputation in Stereo-seq may improve when highly variable gene selection is applied. Stereo-seq is a much larger and sparser dataset compared with 10$\times $ Genomics Visium. This is one of the reasons why gene selection results in better performance.

#### Performance on Slide-seqV2

**Figure 5:**
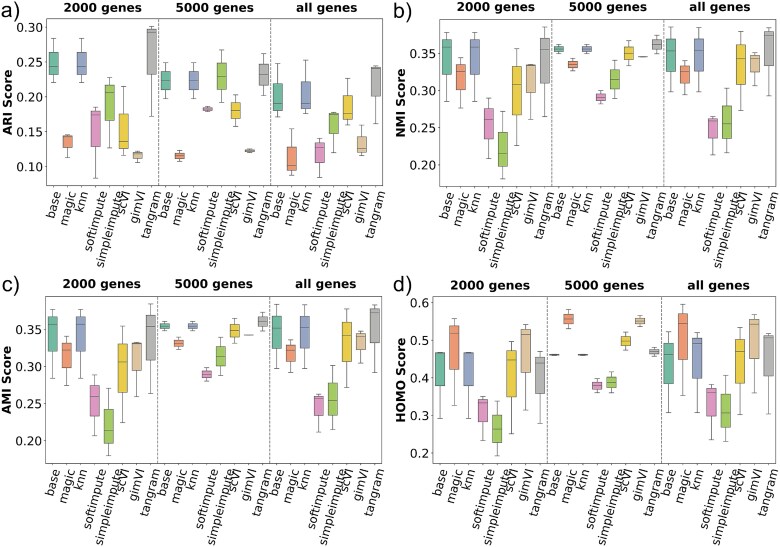
Clustering performance on the Slide-seqV2 dataset across three gene selection levels (2000 genes, 5000 genes, and all genes), evaluated using (a) ARI, (b) NMI, (c) AMI, and (d) HOMO scores. The imputation method, tangram, consistently outperforms all other methods across most metrics and gene selections.

Figure 5 shows clustering performance on the Slide-seqV2. Across all four metrics, **tangram** supersedes all other methods, including the base method. gimVI also performs well for AMI and HOMO scores. However, simpler methods like magic and SimpleImpute show poor performance in most scenarios, indicating that an imputation method performing better in one technology might not perform good for other technologies. In contrast, deep-learning-based methods such as scVI, gimVI, and tangram generally performed well. This may contribute to the fact that Slide-seqV2 is high-dimensional and consists of higher sparsity compared with 10$\times $ Genomics Visium. The HOMO scores highlight that tangram and gimVI can maintain better cluster HOMO in some settings. KNN Impute also performed well. However, it was later discovered that KNN Impute fails to impute such high sparse data effectively. Furthermore, Fig. 5 demonstrates that when 5000 genes are selected, the performance variability across different imputation methods is reduced, as evidenced by the narrower interquartile ranges in the box plots. This observation implies that the stability and consistency of imputation methods improve with the inclusion of a larger set of gene features in the Slide-seqV2.

#### Performance on XYZeq

**Figure 6:**
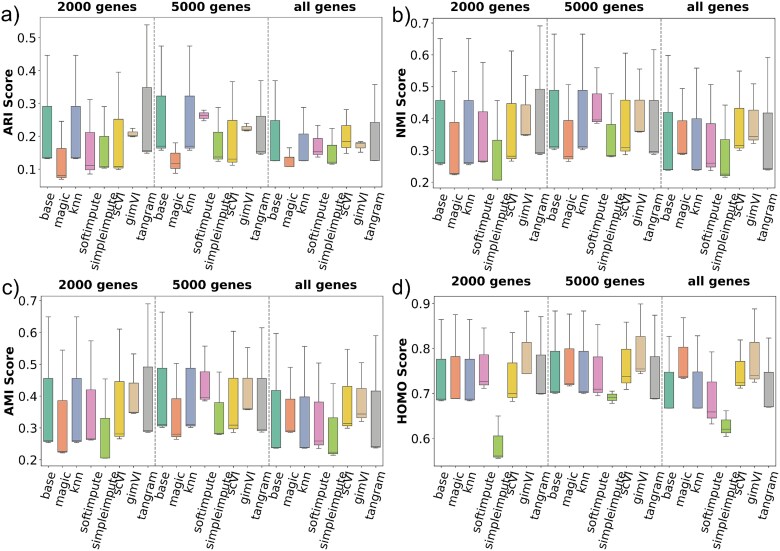
Clustering evaluation results on the XYZeq dataset across three gene selection levels (2000 genes, 5000 genes, and all genes), assessed using (a) ARI, (b) NMI, (c) AMI, and (d) HOMO scores. The boxplot distributions appear skewed in most cases. Deep learning-based methods such as scVI, gimVI, and tangram demonstrate competitive clustering performance across all metrics and gene subsets. In contrast, simpler imputation methods consistently underperform, highlighting the importance of model complexity in handling the characteristics of XYZeq.

Figure 6 presents evaluation results on the XYZeq where the boxplot distribution seems skewed for most cases. While the base method maintains moderate clustering quality, deep learning-based imputation methods such as scVI, gimVI, and tangram show improved performance. However, other simpler methods consistently underperform across all metrics and gene settings.

We also evaluated these imputation methods on the sci-Space dataset. However, we found only one sci-Space dataset having annotations. Hence, we did not generate a box plot distribution for this technology. However, the details of the clustering metrics results for this technology can be found in the Supplementary Document 1, where **MAGIC** consistently outperformed across all metrics and gene selections. This indicates that some technology datasets (for e.g. sci-Space) may need simpler imputation methods, whereas others (for e.g. XYZeq, Stereo-seq, and Slide-seqV2) may need more complex, deep learning-based architectures due to the inherently complex nature of the datasets.

The benchmarking results indicate that no single imputation method consistently outperforms others across all SRT technologies. However, some methods demonstrate superior performance for specific technologies. Furthermore, the results highlight the impact of gene selection on performance, as observed in Stereo-seq and Slide-seqV2, where selecting the top 2000 or 5000 genes leads to more stable and improved clustering outcomes. These findings emphasize the importance of aligning imputation strategies with both the characteristics of the technology and the gene selection criteria.

### Evaluation of dropout reduction capability across imputation methods

Imputation methods must have the ability to differentiate between a valid dropout and a biological zero. Otherwise, it will impute the biological zeros, resulting in the distortion of the underlying biological nature of the datasets. Here, we discuss how these dropouts are removed by methods. Table 2 shows the percentage of zero values in the 10$\times $ Genomics Visium datasets before and after imputation across three different gene selection scenarios. The raw data exhibit extremely high sparsity, with most datasets having over 85%–95% zeros. Imputation methods such as SoftImpute, SimpleImpute, scVI, gimVI, and tangram completely eliminate all zeros, leading to a zero sparsity of 0%. In contrast, MAGIC shows a partial but very high amount of imputation depending on the dataset. Notably, the KNN Impute method does not reduce sparsity at all, maintaining the same percentage of zeros as in the raw data. This indicates that KNN Impute do not have the ability to impute any dropouts. To ensure, the K-value was varied, and zero sparsity remained the same as the raw data. Conversely, SoftImpute and SimpleImpute need prior knowledge of dropouts. Hence, we set all the zeros in the Raw data to “NaN” values that are imputed by these methods. In this way, all the zeros are imputed, whether they are due to dropout or biological reasons. However, the deep learning-based methods do not need prior knowledge, yet they also impute all the zeros, i.e. fail to differentiate the actual dropouts among the zeros in SRTs.

**Table 2:** Percentage of zero sparsity in the 10$\times $ Genomics Visium datasets before (Raw) and after applying imputation methods

SL No.	Top genes	Dataset name	RAW	MAGIC	KNN impute	Soft impute	Simple impute	scVI	gimVI	tangram
01.	2000	151507	93.99	0.021	93.99	0	0	0	0	0
		151508	94.86	0	94.86	0	0	0	0	0
		151509	94.63	0.30	94.63	0	0	0	0	0
		151510	94.72	0.28	94.72	0	0	0	0	0
		151669	89.61	0	89.61	0	0	0	0	0
		151670	88.31	0	88.31	0	0	0	0	0
		151671	91.23	0	91.23	0	0	0	0	0
		151672	92.002	0	92.002	0	0	0	0	0
		151673	93.99	0.019	93.99	0	0	0	0	0
		151674	89.54	0.23	89.54	0	0	0	0	0
		151675	91.64	0.20	91.64	0	0	0	0	0
		151676	91.14	0.05	91.14	0	0	0	0	0
		BRCA1	80.38	0.37	80.38	0	0	0	0	0
02.	5000	151507	92.41	0.003	92.41	0	0	0	0	0
		151508	93.44	0	93.44	0	0	0	0	0
		151509	93.41	0.23	93.41	0	0	0	0	0
		151510	93.07	0.15	93.07	0	0	0	0	0
		151669	86.08	0	86.08	0	0	0	0	0
		151670	85.78	0	85.78	0	0	0	0	0
		151671	89.58	0	89.58	0	0	0	0	0
		151672	89.84	0	89.84	0	0	0	0	0
		151673	92.41	0.005	92.41	0	0	0	0	0
		151674	88.62	0.13	88.62	0	0	0	0	0
		151675	90.28	0.1	90.28	0	0	0	0	0
		151676	89.84	0.03	89.84	0	0	0	0	0
		BRCA1	79.83	0.45	79.83	0	0	0	0	0
03.	all	151507	89.69	0.01	89.69	0	0	0	0	0
		151508	90.88	0	90.88	0	0	0	0	0
		151509	90.48	0.20	90.48	0	0	0	0	0
		151510	90.52	0.16	90.52	0	0	0	0	0
		151669	84.28	0	84.28	0	0	0	0	0
		151670	84.28	0	84.28	0	0	0	0	0
		151671	86.45	0	86.45	0	0	0	0	0
		151672	86.66	0	86.66	0	0	0	0	0
		151673	89.69	0.02	89.69	0	0	0	0	0
		151674	83.94	0.15	83.94	0	0	0	0	0
		151675	87.35	0.12	87.35	0	0	0	0	0
		151676	86.43	0.08	86.43	0	0	0	0	0
		BRCA1	73.21	1.26	73.21	0	0	0	0	0

We observe a similar trend in zero sparsity after imputing the Stereo-seq datasets as shown in Table 3. KNN Impute still fails to impute any data despite different K values, whereas other imputation methods impute all zeros in the datasets. This is a big concern as all the zeros cannot be due to technical reasons. Thus, imputation methods must have a detection capability that can differentiate between true dropout and actual biological expression in SRTs. For other technologies, we have found similar zero-sparsity trends and showed detailed results in the Supplementary Document 1.

**Table 3:** Percentage of zero sparsity in the stereo-seq datasets before (Raw) and after applying imputation methods.

SL No.	Top genes	Dataset name	RAW	MAGIC	KNN impute	Soft impute	Simple impute	scVI	gimVI	tangram
01.	2000	DT2_D0	92.88	0.05	92.88	0	0	0	0	0
		DX6_D2	86.18	0	0.002	86.18	0	0	0	0
		FB2_D1	86.04	0.002	86.04	0	0	0	0	0
02.	5000	DT2_D0	92.5	0.019	92.5	0	0	0	0	0
		DX6_D2	84.92	0.003	84.92	0	0	0	0	0
		FB2_D1	84.62	0.006	84.62	0	0	0	0	0
03.	all	DT2_D0	89.67	0.06	89.67	0	0	0	0	0
		DX6_D2	84.41	0.017	84.41	0	0	0	0	0
		FB2_D1	83.44	0.079	83.44	0	0	0	0	0

Methods such as gimVI and tangram are designed with internal mechanisms to probabilistically distinguish between technical dropouts and biological zeros [24, 25]. For example, gimVI employs a generative model to estimate dropout probability, while tangram leverages dense reference transcriptomes for spatial mapping. However, they completely removed all zeros, including biological ones. This suggests that, despite their theoretical ability, they may not effectively differentiate valid dropouts from biological zeros in practice. Consequently, this over-imputation may distort the biological integrity of the datasets. Overall, the dropout reduction capability demonstrates that all the methods tend to overimpute.

### Computational complexity among the imputation methods

**Figure 7:**
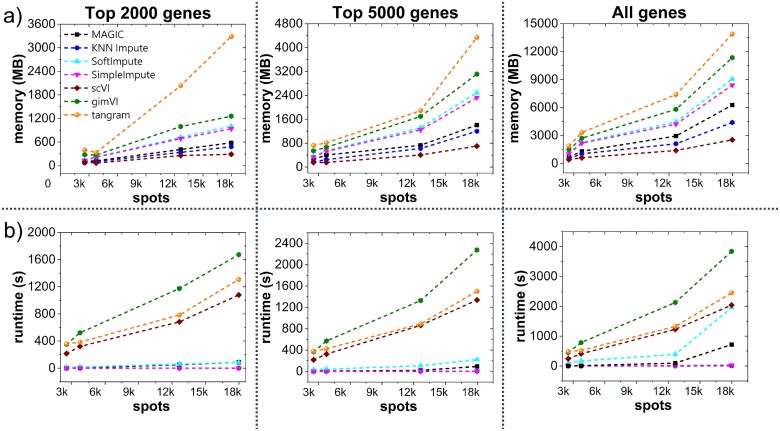
Computational complexity of the imputation methods for varying number of spots (a) Memory consumption and (b) Runtime comparison for the imputation methods for 2000 genes, 5000 genes, or all genes, deep learning-based methods, particularly gimVI and scVI, exhibit the highest runtimes due to their iterative training and complex model architectures, whereas tangram has the highest memory consumption.

In this section, we discuss the computational complexity of the benchmarked imputation methods. We used the NVIDIA A100 GPU available in Colab, typically features 40 GB HBM2 memory, up to 312 TFLOPS FP16 performance, and over 1.5 TB/s memory bandwidth, making it highly suitable for large-scale deep learning and spatial omics workloads. Figure 7 demonstrates the memory and runtime during imputation time vs the number of spots for each imputation method for the different feature subsets. The total of 23 datasets from five technologies have various ranges of spots/cells, with 10$\times $ Genomics Visium having the lowest one (around 3 thousand spots) and Stereo-seq having the highest one (around 20 thousand spots). We can observe from Fig. 7 that the memory and runtime increase with the number of spots. Figure 7a demonstrates that tangram takes maximum memory during imputation, whereas gimVI takes the maximum runtime for imputation (Fig. 7b). Surprisingly, scVI takes the lowest memory, even compared with other simple imputation methods, but has a very high runtime with an increasing number of spots. The observed computational demands of deep learning-based methods—particularly scVI, gimVI, and tangram—can be attributed to their architectural complexity and data handling strategies. Specifically, both scVI and gimVI utilize VAE frameworks involving iterative stochastic optimization over multiple epochs. gimVI, an extension of scVI, incorporates both spatial and gene expression modalities, adding further computational burden during training, which explains its higher runtime compared with scVI. Despite the high runtime, scVI exhibits surprisingly low memory usage. This can be attributed to its use of mini-batch training and efficient memory management practices in PyTorch, which allow it to process subsets of the data sequentially rather than loading the full dataset into memory. In contrast, gimVI requires access to both paired spatial and transcriptomic inputs that increases the dimensionality and memory footprint of each training sample. Moreover, it often disables aggressive mini-batching due to spatial dependencies, leading to higher memory use. tangram, on the other hand, requires memory-intensive matrix operations and alignment steps between single-cell and SRT datasets. These operations are often dense and require holding large matrices in memory, especially when aligning high-dimensional single-cell reference data to tens of thousands of spatial locations. As a result, tangram shows high memory usage even though its runtime is not as extreme as gimVI. In comparison, traditional methods such as magic, knn, or simpleimpute have simpler algorithmic steps, resulting in moderate increases in runtime and memory as the dataset size grows. However, their computational load is negligible compared with deep learning-based methods that are designed for high-capacity learning but come at a significant computational cost. Other basic imputation methods also take higher runtime and memory with an increasing number of spots; however, they are negligible compared with the deep-learning methods.

Although a distinct generalizable pattern was not discovered from our evaluation framework, some patterns can be established according to the RQs and results generated from the benchmark. Table 4 summarizes the results to provide insights into any general patterns across imputation methods and their performance. First, no imputation method universally dominates; rather, performance is strongly technology-dependent. Second, the complexity of an imputation method should match the sparsity and dimensionality of the dataset. Simple approaches (e.g. MAGIC) can outperform deep models, while deep-learning methods (e.g. tangram, gimVI, and scVI) excel on high-dimensional, highly sparse data (e.g. Slide-seqV2 and XYZeq). Third, gene selection strongly influences performance: “all genes” benefits denser technologies like 10$\times $ Genomics Visium, while top-variable-gene subsets improve stability and accuracy for sparse technologies like Stereo-seq. Fourth, all evaluated methods impute nearly all zeros, including probable biological zeros, underscoring a field-wide gap in distinguishing technical dropouts from true biological zeros. Finally, deep-learning models incur significantly higher runtime and memory costs compared with simpler methods. Collectively, these patterns underscore the value of our evaluation framework for guiding technology-specific imputation choices, gene-selection strategies, and balancing biological fidelity with computational feasibility.

**Table 4:** Summary of the results discovered in the benchmark pipeline associated with RQs

Dimension	Systematic insight	Evidence from results	Implication for evaluation framework
Across technologies (10$\times $ Genomics Visium, Stereo-seq, Slide-seqV2, and XYZeq, sci-Space)	No single imputation method consistently outperforms across all SRT platforms.	gimVI best for Visium; tangram for Slide-seqV2; MAGIC for sci-Space and Stereo-seq.	Choice of imputation must be technology-specific rather than “one-size-fits-all.”
Method complexity (simple vs deep learning)	Simple methods sometimes outperform deep models on low-complexity or less sparse data; deep models excel on high-dimensional, highly sparse data.	MAGIC and base method outperform deep models in Stereo-seq (less stable for deep models); tangram/gimVI/scVI outperform in Slide-seqV2 and XYZeq.	The framework shows that method complexity should be aligned with data sparsity and dimensionality.
Gene selection effect	Selecting the top 2000–5000 genes often improves clustering stability and performance for highly sparse datasets; using all genes benefits denser datasets.	In 10$\times $ Genomics Visium, “all genes” yields better ARI/NMI/AMI; in Stereo-seq and Slide-seqV2, 2000–5000 genes yield more stable clustering and narrower IQRs.	Gene-selection strategy critically influences performance; evaluation must include multiple gene-set sizes.
Zero handling/dropout vs biological zeros	All tested methods (except KNN) impute nearly 100% of zeros, regardless of whether technical or biological, suggesting over-imputation.	Results of zero sparsity show 0% zeros after imputation for deep methods and SoftImpute/SimpleImpute; KNN retains original zeros.	Framework reveals a gap in current methods: inability to distinguish dropouts from true biological zeros.
Computational cost vs performance	Deep-learning methods, in general, achieve better performance in complex datasets but incur heavy runtime and/or memory costs; simple methods scale efficiently but perform poorly in high-sparsity settings.	tangram has the highest memory; gimVI highest runtime; scVI lowest memory but long runtime.	Evaluation should consider the computational cost alongside biological accuracy to balance practical applicability.
Technology-driven method suitability	Sparser/larger datasets (Stereo-seq, Slide-seqV2, and XYZeq) benefit from deep models; less sparse or lower-dimensional datasets (10$\times $ Genomics Visium, sci-Space) perform equally or better with simpler methods or even no imputation.	Observed in technology-specific benchmarking across all metrics.	Imputation strategies must be technology-aware; this supports the value of the evaluation framework.

### Performance of the SpaMean-Impute

We proposed SpaMean-Impute considering two facts: (i) utilization of spatial information in SRT datasets while imputing, (ii) detection of valid dropouts. Considering these criteria, we tested our proposed method on the different datasets and found that our method outperformed SOTA imputation methods, demonstrating stable performance. Although integration of spatial information in SRTs imputation is common [24, 25, 27, 28], valid dropout detection is missing in the literature. Our method can detect valid dropouts and hence preserve the actual zeros, unlike the SOTA methods’ inability to distinguish between valid dropouts and actual zeros as discussed in Section “Evaluation of dropout reduction capability across imputation methods.” Hence, the enhancement of clustering metrics can be achieved with imputing valid dropouts. This also reflects in the zero sparsity before and after imputation using our method. In the following sections, the performance of the proposed method is discussed on the SRT technologies, excluding XYZeq technology due to the lack of spatial location information in the XYZeq dataset we acquired.

#### Comparison of clustering metrics of SpaMean-Impute with state-of-the-art methods

As we demonstrated in our benchmarking results (Section “Evaluation of imputation methods using clustering-based metrics”), highly variable genes have a significant impact on the imputation methods’ performance and subsequently on the clustering results. For example, imputation methods perform well when all genes are selected for 10$\times $ Genomics Visium, whereas top 2000 or 5000 genes selection work well in the case of Stereo-seq, Slide-seqV2, and sci-Space. Hence, we considered all genes for 10$\times $ Genomics Visium, top 2000 genes for Stereo-seq, and top 5000 genes for Slide-seqV2 when comparing our proposed method’s performance with SOTA methods.

Table 5 presents the ARI scores across SRTs compared with ‘SpaMean-Impute’. Across the 10$\times $ Genomics Visium datasets, SpaMean-Impute consistently achieves the highest ARI scores for all samples, significantly outperforming both baseline and existing methods. In the case of Stereo-seq data, although MAGIC occasionally performs competitively, SpaMean-Impute still provides superior performance across all samples. For Slide-seqV2, which typically presents more sparsity and complexity, SpaMean-Impute again outperforms other methods, including scVI and tangram, showing its adaptability across gene densities and platform characteristics. Overall, this table illustrates that SpaMean-Impute not only generalizes well across technologies and gene selection settings, but also consistently leads to better clustering fidelity.

**Table 5:** ARI scores for SOTA imputation methods compared with “SpaMean-Impute”

SL No.	Technology	Top genes	Dataset name	base	MAGIC	KNN impute	Soft impute	Simple impute	scVI	gimVI	tangram	SpaMean-Impute
01.	10$\times $ Genomics Visium	all	151507	0.26	0.18	0.27	0.06	0.19	0.18	0.22	0.24	**0.36**
			151508	0.25	0.17	0.25	0.08	0.14	0.17	0.19	0.20	**0.31**
			151509	0.21	0.10	0.21	0.12	0.23	0.17	0.16	0.20	**0.30**
			151510	0.27	0.12	0.27	0.09	0.17	0.16	0.24	0.22	**0.32**
			151669	0.40	0.17	0.38	0.09	0.23	0.19	0.23	0.34	**0.45**
			151670	0.36	0.16	0.36	0.07	0.22	0.19	0.26	0.26	**0.49**
			151671	0.47	0.24	0.47	0.17	0.30	0.31	0.29	0.41	**0.49**
			151672	0.45	0.22	0.44	0.10	0.28	0.33	0.28	0.37	**0.48**
			151673	0.26	0.19	0.27	0.07	0.19	0.18	0.21	0.24	**0.29**
			151674	0.33	0.19	0.33	0.17	0.24	0.28	0.23	0.25	**0.35**
			151675	0.44	0.25	0.42	0.23	0.35	0.28	0.34	0.40	**0.54**
			151676	0.37	0.23	0.38	0.23	0.26	0.28	0.29	0.35	**0.41**
			BRCA1	0.18	0.08	0.18	0.14	0.19	0.14	0.12	0.17	**0.19**
02.	Stereo-seq	2000	DT2_D0	0.26	0.29	0.26	0.26	0.31	0.19	0.24	0.12	**0.31**
			DX6_D2	0.27	0.27	0.27	0.12	0.18	0.19	0.22	0.15	**0.29**
			FB2_D1	0.21	0.23	0.21	0.10	0.17	0.13	0.14	0.09	**0.23**
03.	Slide-seqV2	5000	WT	0.24	0.12	0.24	0.18	0.26	0.20	0.12	0.26	**0.27**
			diabetes	0.19	0.10	0.19	0.17	0.19	0.15	0.12	0.20	**0.22**
			mouse	0.19	0.15	0.19	0.08	0.11	0.15	0.15	0.16	**0.20**
04.	sci-Space	5000	GSE 166692	0.45	0.41	0.45	0.24	0.34	0.43	0.37	0.44	**0.48**

The highest score per row is bolded.

Furthermore, Table 6 reports the comparison for NMI scores where SpaMean-Impute consistently achieves the highest NMI scores, with values reaching up to 0.67 for dataset 151675 for 10$\times $ Genomics Visium. It outperforms methods such as SoftImpute, SimpleImpute, and deep learning models like scVI, gimVI, and tangram. Similar performance is observed for sci-Space technology. The NMI scores for SpaMean-Impute remain notably superior across most samples, indicating its robustness in preserving mutual information between predicted and ground truth labels. In the Stereo-seq datasets, although MAGIC briefly matches the top performance in one instance (e.g. DX6_D2), SpaMean-Impute again generally shows stable performance, suggesting adaptability across varying spatial resolution and sparsity. Although Table 5 shows that SpaMean-Impute performs competitively on the Stereo-seq datasets in terms of ARI, its performance in terms of NMI is relatively lower. This discrepancy arises from the intrinsic differences between these two clustering evaluation metrics. The ARI metric evaluates clustering accuracy by comparing all pairs of samples and checking how consistently they are grouped in both the predicted and true labels. It is particularly sensitive to the precise grouping of sample pairs and penalizes random agreements. In contrast, NMI measures the amount of mutual information shared between predicted and true labels, normalized by their entropies. NMI tends to reward clusterings that align well in terms of label distributions and balanced entropy, even if some sample groupings are slightly off. In the case of Stereoseq, which exhibits high data sparsity, SpaMean-Impute appears effective at correctly assigning sample pairs to the same clusters, yielding high ARI values. However, the predicted cluster distributions might not align as well in entropy or size balance with the true labels, leading to comparatively lower NMI scores. Similarly, for Slide-seqV2 data, which tends to be more sparse and noisy, SpaMean-Impute achieves the best NMI values. Overall, the results highlight the advantage of spatially aware, tailored imputation like SpaMean-Impute for enhancing downstream clustering outcomes.

**Table 6:** Comparison of NMI Scores with “SpaMean-Impute”

SL No.	Technology	Top genes	Dataset name	base	MAGIC	KNN impute	Soft impute	Simple impute	scVI	gimVI	tangram	SpaMean-Impute
01.	10$\times $ Genomics Visium	all	151507	0.43	0.38	0.44	0.161	0.32	0.32	0.40	0.39	**0.55**
			151508	0.40	0.35	0.40	0.19	0.28	0.31	0.36	0.36	**0.52**
			151509	0.40	0.34	0.39	0.25	0.39	0.36	0.39	0.376	**0.53**
			151510	0.43	0.34	0.43	0.21	0.28	0.29	0.43	0.38	**0.52**
			151669	0.43	0.31	0.43	0.17	0.31	0.24	0.36	0.39	**0.53**
			151670	0.43	0.31	0.42	0.15	0.33	0.26	0.37	0.40	**0.53**
			151671	0.52	0.39	0.52	0.29	0.36	0.36	0.45	0.50	**0.59**
			151672	0.54	0.41	0.54	0.24	0.41	0.39	0.47	0.50	**0.60**
			151673	0.43	0.40	0.44	0.15	0.32	0.33	0.40	0.40	**0.47**
			151674	0.46	0.42	0.46	0.29	0.38	0.37	0.43	0.40	**0.52**
			151675	0.53	0.48	0.50	0.38	0.47	0.43	0.55	0.49	**0.67**
			151676	0.52	0.45	0.53	0.34	0.41	0.41	0.49	0.48	**0.60**
			BRCA1	0.37	0.31	0.37	0.32	0.37	0.31	0.31	0.37	**0.39**
02.	Stereo-seq	2000	DT2_D0	0.40	0.46	0.40	0.38	0.46	0.33	0.46	0.33	**0.48**
			DX6_D2	0.40	**0.46**	0.40	0.22	0.29	0.29	0.36	0.24	0.43
			FB2_D1	0.30	**0.34**	0.30	0.18	0.27	0.20	0.24	0.14	0.32
03.	Slide-seqV2	5000	WT	0.36	0.34	0.36	0.29	0.34	0.36	0.34	0.37	**0.38**
			diabetes	0.34	0.32	0.34	0.28	0.28	0.33	0.34	0.34	**0.353**
			mouse	0.29	0.29	0.29	0.21	0.21	0.27	0.30	0.29	**0.30**
04.	sci-Space	5000	GSE 166692	0.67	0.68	0.67	0.5	0.58	0.65	0.63	0.67	**0.70**

The highest score per row is bolded.

Table 7 demonstrates the AMI comparative scores. Among the 10$\times $ Genomics Visium datasets, SpaMean-Impute consistently achieves the highest AMI scores, demonstrating significant improvements over baseline and existing imputation techniques. For Stereo-seq, while MAGIC performs competitively, achieving the highest score on DX6_D2 and FB2_D1, SpaMean-Impute leads on DT2_D0, indicating effectiveness depending on the sample. On the more sparse and noisy Slide-seqV2 datasets, AMI scores are closely clustered among methods, with SpaMean-Impute providing marginal gains. Lastly, in the sci-Space dataset, SpaMean-Impute outperforms all baselines, achieving the top AMI score. These results reinforce that SpaMean-Impute not only generalizes across technological platforms but also offers reliable clustering accuracy as reflected in mutual information metrics.

**Table 7:** AMI scores for SOTA imputation methods compared with “SpaMean-Impute”

SL No.	Technology	Top genes	Dataset name	base	MAGIC	KNN impute	Soft impute	Simple impute	scVI	gimVI	tangram	SpaMean-Impute
01.	10$\times $ Genomics Visium	all	151507	0.43	0.38	0.43	0.15	0.31	0.32	0.4	0.39	**0.55**
			151508	0.4	0.34	0.4	0.18	0.28	0.3	0.35	0.35	**0.52**
			151509	0.39	0.33	0.39	0.24	0.39	0.35	0.39	0.37	**0.54**
			151510	0.43	0.33	0.42	0.21	0.28	0.29	0.43	0.38	**0.52**
			151669	0.43	0.3	0.43	0.17	0.31	0.24	0.35	0.39	**0.55**
			151670	0.43	0.3	0.41	0.14	0.33	0.25	0.37	0.4	**0.52**
			151671	0.52	0.39	0.52	0.28	0.35	0.36	0.45	0.50	**0.59**
			151672	0.54	0.40	0.54	0.24	0.41	0.39	0.47	0.50	**0.59**
			151673	0.43	0.39	0.43	0.15	0.31	0.33	0.39	0.40	**0.45**
			151674	0.46	0.42	0.46	0.29	0.38	0.37	0.42	0.40	**0.52**
			151675	0.53	0.47	0.50	0.38	0.47	0.42	0.55	0.49	**0.67**
			151676	0.52	0.44	0.52	0.34	0.40	0.41	0.48	0.47	**0.60**
			BRCA1	0.37	0.31	0.37	0.32	0.37	0.31	0.31	0.37	**0.38**
02.	Stereo-seq	2000	DT2_D0	0.40	0.46	0.40	0.38	0.46	0.33	0.38	0.19	**0.48**
			DX6_D2	0.40	**0.46**	0.40	0.22	0.29	0.29	0.36	0.24	0.36
			FB2_D1	0.30	**0.34**	0.30	0.18	0.27	0.20	0.24	0.13	0.3
03.	Slide-seqV2	5000	WT	0.36	0.33	0.36	0.29	0.33	0.36	0.34	**0.37**	0.34
			diabetes	0.34	0.32	0.34	0.28	0.28	0.33	0.34	0.34	**0.35**
			mouse	0.29	0.29	0.29	0.21	0.21	0.27	**0.3**	0.29	0.29
04.	sci-Space	5000	GSE 166692	0.67	0.67	0.67	0.5	0.57	0.64	0.62	0.66	**0.69**

The highest score per row is bolded.

Furthermore, across the 10$\times $ Genomics Visium datasets, SpaMean-Impute consistently achieved the highest HOMO scores, surpassing both conventional imputation baselines and deep learning approaches, thus indicating a strong preservation of class-specific structure post-imputation (Table 8). For the sci-Space dataset, SpaMean-Impute again achieved the top HOMO score. In contrast, for datasets from the Stereo-seq and Slide-seqV2 platforms, the HOMO scores for all methods—including SpaMean-Impute, are generally lower. This can be attributed to the higher sparsity and noisier gene expression profiles inherent to these technologies, which pose significant challenges for accurately reconstructing spot type boundaries. Moreover, due to the ultra-high spatial resolution in these platforms, spatial spots often capture only partial transcriptomic profiles or contain mixed cell signals, leading to degraded clustering HOMO post-imputation. In such contexts, while SpaMean-Impute does not always achieve the best HOMO score, it remains competitive and still offers improvements over other methods.

**Table 8:** Comparison of HOMO scores with “SpaMean-Impute”

SL No.	Technology	Top genes	Dataset name	base	MAGIC	KNN impute	Soft impute	Simple impute	scVI	gimVI	tangram	SpaMean-Impute
01.	10$\times $ Genomics Visium	all	151507	0.45	0.46	0.45	0.17	0.32	0.36	0.48	0.42	**0.61**
			151508	0.42	0.42	0.41	0.2	0.27	0.35	0.41	0.38	**0.57**
			151509	0.44	0.46	0.43	0.28	0.42	0.45	0.5	0.41	**0.62**
			151510	0.4	0.44	0.46	0.24	0.28	0.35	0.52	0.43	**0.57**
			151669	0.46	0.4	0.5	0.2	0.38	0.29	0.43	0.45	**0.59**
			151670	0.5	0.43	0.48	0.19	0.41	0.32	0.47	0.51	**0.6**
			151671	0.56	0.52	0.56	0.34	0.4	0.43	0.59	0.58	**0.64**
			151672	0.6	0.55	0.59	0.3	0.5	0.47	0.64	0.6	**0.67**
			151673	0.45	0.49	0.45	0.16	0.32	0.36	0.46	0.42	**0.49**
			151674	0.5	0.55	0.5	0.34	0.43	0.41	0.52	0.46	**0.58**
			151675	0.53	0.59	0.54	0.46	0.49	0.5	0.66	0.52	**0.74**
			151676	0.57	0.57	0.58	0.4	0.43	0.47	0.59	0.53	**0.66**
			BRCA1	0.57	0.55	0.57	0.52	0.57	0.47	0.5	0.57	**0.61**
02.	Stereo-seq	2000	DT2_D0	0.32	**0.47**	0.32	0.34	0.39	0.30	0.37	0.19	0.38
			DX6_D2	0.34	**0.44**	0.34	0.21	0.25	0.25	0.34	0.23	0.31
			FB2_D1	0.26	**0.34**	0.26	0.17	0.26	0.18	0.25	0.13	0.27
03.	Slide-seqV2	5000	WT	0.46	**0.58**	0.46	0.39	0.41	0.52	0.56	0.48	0.44
			diabetes	0.45	**0.53**	0.45	0.36	0.35	0.47	**0.53**	0.45	0.47
			mouse	0.3	**0.35**	0.3	0.23	0.23	0.3	**0.35**	0.3	0.3
04.	sci-Space	5000	GSE 166692	0.63	0.69	0.63	0.44	0.51	0.6	0.58	0.62	**0.74**

The highest score per row is bolded.

According to the overall clustering results, it is evident that “SpaMean-Impute” demonstrates consistently competitive performance across all evaluation metrics. Specifically, it achieves relatively high values in ARI, NMI, and AMI, indicating its effectiveness in aligning predicted clusters with true labels. This suggests that SpaMean-Impute preserves biological structure while maintaining cluster purity and label consistency after imputation compared with other SOTA methods. Furthermore, the SpaMean-Impute was also applied to a recent Visium Datasets [53] to explore its effectiveness after imputation on more advanced technology. It was observed that SpaMean-Impute consistently performed across these evaluation metric as well for this recent dataset. The details can be found in Supplementary Document 1 (Section S7).

Additionally, since our imputation algorithm averages transcriptome profiles of spatially adjacent and transcriptionally similar cells, an assumption that may risk a potential circularity problem if performance is only judged by clustering similarity. To address this, we performed an independent test by masking a portion of non-zero entries (1%–10%) and evaluating how well the method recovered the true values. Across multiple spatial transcriptomics platforms (Slide-seqV2, 10$\times $ Genomics Visium, Stereo-seq, and sci-Space), the algorithm consistently recovered a substantial proportion of masked values (e.g. 17%–20% in 10$\times $ Genomics Visium, $\sim $72%–77% in Stereo-seq) and maintained moderate-to-high agreement with the ground truth, as measured by Pearson Correlation Coefficient (PCC $\sim $0.60–0.84), Spearman Rank Correlation (SRC $\sim $0.58–0.85), and Kendall’s Tau Correlation (KTC $\sim $0.57–0.81). These results confirm that our model truly predicts missing transcriptomic values rather than merely reinforcing spatial similarity. The detail results are provided in the Supplementary Document 1 (Section S8).

#### Average zero sparsity percentage comparison of SpaMean-Impute

SpaMean-Impute consider the zero values to be dropout based on the nearest neighbor and their corresponding values of the respective genes. Hence, this method does not impute the biological zeros as demonstrated in Table 9. As discussed earlier, it can be observed again that the average zero values become completely imputed after using the seven imputation methods. However, when SpaMean-Impute is applied to the RAW data, the average zero values after the imputation process still remain pretty high, on average 85.6% zeros as valid zeros, indicating 0.85% to be the dropout and imputed them. In the previous section “Comparison of clustering metrics of SpaMean-Impute with SOTA methods,” it was demonstrated how this proposed method outperformed significantly considering this small proportion to be dropped out. SpaMean-Impute found a higher dropout in Stereo-seq, whereas on average 0.5%–0.54% in case of Slide-seqV2 and sci-Space. However, the parameters nearest-neighbor, $k$, and $\delta $ (dropout threshold) play crucial roles in controlling the balance between sensitivity and specificity of dropout recovery. A higher $k$ value increases the spatial context by considering more neighbors during imputation. While this may improve robustness to noise, it may also introduce averaging effects that dilute local expression patterns. Conversely, a smaller $k$ may better preserve local heterogeneity but risks being more sensitive to local outliers or noise. The dropout threshold $\delta $ governs whether a zero entry should be considered a dropout or a true biological zero. A lower $\delta $ value makes the method conservative, imputing fewer zeros—thus reducing the risk of falsely imputing genuine non-expression values. In contrast, a higher $\delta $ increases the number of zeros that qualify for imputation, which can enhance data recovery but may also introduce bias if truly non-expressed genes are inappropriately filled. Therefore, fine-tuning $k$ and $\delta $ is essential for optimal imputation performance and should be guided by the sparsity level and biological complexity of the dataset. In our hyperparameter tuning, we observed that the model performs well when $k$ is in the range of 3–11 and $\delta $ in the range of 4%–40%. The details of the hyperparameters sensitivity analysis are provided in the Supplementary Document 1 (Section S6).

**Table 9:** Average zero sparsity percentage comparison of “SpaMean-Impute” with other SOTA imputation methods

SL No.	Technology	Top genes	Zero sparsity in RAW data (%)	After MAGIC (%)	After KNN impute (%)	After soft impute (%)	After simple impute (%)	After scVI (%)	After gimVI (%)	After tangram (%)	After SpaMean-Impute (%)	Dropout detected by SpaMean-Impute (%)
01.	10$\times $ Genomics Visium	all	86.45	0.154	86.45	0	0	0	0	0	85.6	0.85
02.	Stereo-seq	2000	88.37	0.02	88.37	0	0	0	0	0	85.94	2.43
03.	Slide-seqV2	5000	94.51	1.05	94.51	0	0	0	0	0	94.01	0.5
04.	sci-Space	5000	87.65	4.64	87.65	0	0	0	0	0	87.11	0.54

#### Computational complexity of SpaMean-Impute

**Figure 8:**
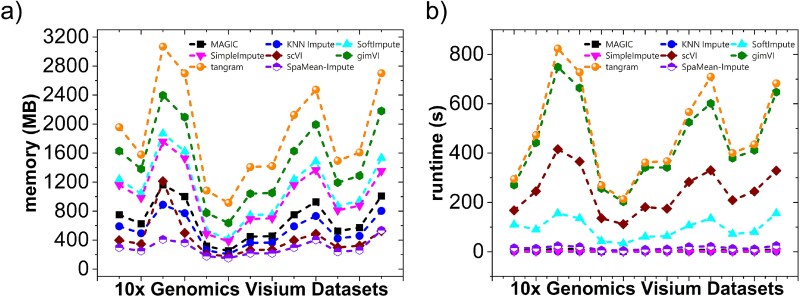
Computational Complexity comparison between SpaMean-Impute and other SOTA methods for 10$\times $ Genomics Visium datasets. (a) Memory consumption and (b) runtime considering all genes. SpaMean-Impute shows one of the lowest runtimes and memory consumption compared with other methods, especially the deep learning-based models.

We also evaluated the memory consumption and runtime of “SpaMean-Impute.” As illustrated in Fig. 8, SpaMean-Impute demonstrates notable computational efficiency across the 10$\times $ Genomics Visium datasets. Specifically, in terms of memory usage (Fig. 8a), SpaMean-Impute maintains consistently low consumption, performing significantly better than deep learning-based models like gimVI and tangram, and comparably or better than simpler methods such as MAGIC and SimpleImpute. Additionally, the runtime comparison (Fig. 8b) shows that SpaMean-Impute is among the fastest methods across all datasets, achieving one of the lowest execution times. Similar observations were found in the case of Stereo-seq, Slide-seqV2, and sci-Space. The details on these technologies are provided in the Supplementary Document 1. This highlights the method’s suitability for large-scale SRT data where both memory and time efficiency are crucial.

The overall performance of SpaMean-Impute indicates that it outperforms all imputation methods utilizing the spatial information in the SRT datasets while showing significantly lower memory consumption and runtime. Compared with the existing approaches like scVI, gimVI, tangram, or other recent approaches, which rely on deep generative models, reference single-cell datasets, or diffusion-based modeling, SpaMean-Impute directly leverages local spatial neighborhoods for statistical imputation. While simpler and more interpretable, it effectively distinguishes true biological zeros from dropouts and preserves spatial consistency, complementing more complex frameworks. However, the proposed method has limitations that include its lower performance when other gene selection settings are selected for different datasets. At present, our proposed method remains a simple statistical model that only imputes using the mean value of its neighbors. Furthermore, the dropout location is only derived using the spatial information. This framework is proposed to imply that integrating spatial information explicitly improves the quality of the SRT datasets. Furthermore, it is clear that the imputation framework of high-dimensional biological data requires several stages, and previous works had employed various deep learning models, such as autoencoders, Graph-based methods, etc., in these stages for improving the quality. We believe that the overall downstream results can be improved if deep-learning-based building blocks are used in these stages. Integration of SOTA deep learning models along with the statistical approach proposed in this study can capture complex nonlinear relationships and spatial dependencies inherent in spatial transcriptomics data. For example, Graph-based deep learning frameworks, such as GNNs and GATs, offer the potential to learn both the spatial topology and gene expression dynamics in a data-driven manner. Additionally, transformer architectures (particularly those enhanced with attention mechanisms) can be employed to dynamically prioritize informative spatial neighbors or contextual patterns during the imputation process. Probabilistic models, such as VAEs and Bayesian neural networks, could also be explored to explicitly model uncertainty in gene dropout events, along with the statistical approach employed currently, and better infer biologically meaningful missing values. Combining these techniques with contrastive or self-supervised learning could enable robust performance even in low-data or noisy scenarios. Finally, tools like autoencoders can be utilized to remove technical biases from SRT datasets and further enhance while imputing the valid dropouts with the help of the present framework.

Recent approaches are also integrating multi-modal approaches for imputing biological data [54–58]. Following these approaches, our future SRT imputation framework can be extended by integrating similar multi-modal approach for enhanced imputation and downstream analysis.

## Conclusion

SRT technologies have emerged as powerful tools that bridge spatial context and transcriptomic profiling, enabling in-depth understanding of tissue architecture and cellular heterogeneity. Recent advances in spatial transcriptomics provide concrete examples of clinical and translational utility. For instance, application of the 10$\times $ Genomics Visium platform to human DLPFC produced layer-specific maps that enriched disease-risk genes for Autism Spectrum Disorder (ASD), schizophrenia, and Alzheimer's disease (AD), where a spatial-registration approach is introduced that anchors dissociated single-cell data to anatomical layers, thereby improving interpretation of clinical single-cell cohorts [35] . Stereo-seq generated a high-definition spatiotemporal liver atlas and identified TBL1XR1 as a regeneration-linked regulator whose *in vivo* knockdown impaired hepatocyte proliferation, directly linking spatial discovery to a validated functional target [37]. Slide-seqV2’s near-cellular sensitivity uncovered dendritically localized RNAs and developmental-disorder gene expression programs, enabling *in situ* detection of low-abundance, spatially critical transcripts, and facilitating RNA-velocity-based reconstruction of developmental axes [38]. Similarly, sci-Space demonstrated single-cell, embryo-scale spatial mapping that quantified how much positional context shapes gene expression and revealed migration dynamics relevant to developmental pathologies [39]. Finally, the XYZeq study, applied to murine tumor models (liver and spleen) using spatially barcoded scRNA-seq, revealed how cellular states (e.g. of mesenchymal stem cells) vary in relation to spatial proximity to the tumor core [40]. Collectively, these case studies show that spatial methods can localize disease biology to precise tissue microenvironments, produce experimentally testable targets, and temporal windows for intervention. These strengthen the translational bridge from discovery to clinic. However, the near single-cell resolution achieved by modern SRT technologies often comes at the cost of high sparsity and dropout events in the gene expression matrices, posing significant challenges for downstream analyses such as clustering and spatial domain identification. In this study, we addressed this critical issue through a comprehensive benchmarking of SOTA imputation methods across 23 datasets from five prominent SRT platforms, including 10$\times $ Genomics Visium, Stereo-seq, Slide-seqV2, XYZeq, and sci-Space.

Our benchmarking reveals that no single existing method consistently excels across all datasets and evaluation metrics. Simpler methods (e.g. MAGIC and SimpleImpute) show limited improvements and often degrade performance under high-sparsity conditions. Deep learning-based models like scVI, gimVI, and tangram demonstrate better performance in certain settings, particularly for highly sparse datasets such as Slide-seqV2; however, they incur significantly higher runtime and memory usage due to their complex model architectures. Notably, scVI exhibits the longest runtime while consuming the least memory, whereas gimVI and tangram are both memory-intensive, reflecting different trade-offs in model design and spatial integration strategies. Additionally, KNN impute fails to impute properly, while other methods impute all the zeros.

Motivated by these findings, we introduced SpaMean-Impute, a novel imputation approach tailored for SRT data that explicitly incorporates spatial information and includes dropout detection. Unlike other methods, SpaMean-Impute distinguishes between technical and biological zeros, reducing the risk of over-imputation. Our method achieves superior performance in most scenarios across all evaluation metrics while maintaining low computational overhead. Particularly, in datasets from 10$\times $ Genomics Visium, Stereo-seq and sci-Space, SpaMean-Impute consistently outperforms all other imputation methods, including the base data, thereby enhancing clustering fidelity and spatial HOMO.

In conclusion, our study underscores the importance of leveraging spatial context in the imputation of SRT data and highlights the limitations of applying generic or scRNA-seq-specific methods in this domain. SpaMean-Impute offers a balanced, accurate, and efficient solution for robust SRT data analysis. Future work could explore integrating deep-learning-based techniques to differentiate between dropouts and biological zeros with proper reasoning in spatial genomics.

**Key Points**

- Single-cell RNA, spatially resolved transcriptomics (SRT)-based imputation, and general imputation methods are benchmarked on five different spatial transcriptomic technologies.
- Performance of the imputation methods shows inconsistent performance.
- Proposed a spatial aware imputation method with dropout detection ability for SRT datasets.
- Integration of spatial data in imputation improves the imputation capability significantly.

## Supplementary Material

ST_breifings_supplementary_V2_bbag027_new

## Data Availability

The current implementation of our work and preprocessed datasets are available at https://github.com/FahimHafiz/SpaMean-Impute.
