# Supplementary material for: Spatial information matters: are traditional imputation methods effective for spatial transcriptomics data?
Source: Brief Bioinform. 2026 Feb 2;27(1):bbag027. doi: 10.1093/bib/bbag027 (PMC12862982; doi:10.1093/bib/bbag027)
Supplement: ST_breifings_supplementary_V2_bbag027_new [file st_breifings_supplementary_v2_bbag027_new.pdf]

Supplementary Materials

Spatial Information Matters: Are Traditional Imputation Methods Effective for Spatial Transcriptomics Data?

Fahim Hafiz[1], Riasat Azim[1], and Swakkhar Shatabda*[2]

[1]Department of Computer Science and Engineering, United International University, Madani Avenue, Dhaka-1212, Bangladesh

[2]Department of Computer Science and Engineering, Brac University, Pragati Sarani, Dhaka-1212, Bangladesh

---

Contents

S1 Detailed Performance on sci-Space **3**

S2 Sparsity in Slide-seqV2 before and after applying Imputation **4**

S3 Sparsity in XYZeq before and after applying Imputation **5**

S4 Sparsity in sci-Space before and after applying Imputation **6**

S5 Computational Performance of 'SpaMean-Impute' on SRT Technologies (excluding 10x Genomics Visium) **7**

S6 Sensitivity Analysis of 'SpaMean-Impute' **9**

*Corresponding Author: swakkhar.shatabda@bracu.ac.bd

S7 Performance of ‘SpaMean-Impute’ on recent SRT Datasets 10

S8 Independent Test for True Signal Recovery for the proposed method 11

S1 Detailed Performance on sci-Space

The benchmark performance of the imputation methods based on ARI, NMI, AMI, and HOMO scores is presented in Table S1. We can observe that the MAGICoutperformed all the methods, especially for the 5000 or all genes case.

Table S1: Performance metrics for different imputation methods applied to sci-Space across varying numbers of top genes

Dataset Name	Metric	Top Genes	base	MAGIC	KNN Impute	Soft Impute	Simple Impute	scVI	gimVI	tan gram
GSE 166692	ARI	2000	0.39	**0.44**	0.39	0.24	0.26	0.39	0.38	0.39
		5000	**0.45**	0.41	**0.45**	0.24	0.35	0.43	0.38	0.44
		all	0.35	**0.47**	0.35	0.22	0.22	0.40	0.41	0.32
	NMI	2000	0.66	**0.68**	0.66	0.49	0.52	0.61	0.62	0.65
		5000	0.67	**0.68**	0.671	0.51	0.58	0.65	0.63	0.672
		all	0.60	**0.70**	0.60	0.42	0.44	0.65	0.65	0.59
	AMI	2000	0.65	**0.67**	0.65	0.49	0.51	0.60	0.61	0.64
		5000	0.66	**0.68**	0.67	0.50	0.57	0.64	0.62	0.66
		all	0.59	**0.69**	0.59	0.41	0.43	0.64	0.63	0.58
	HOMO	2000	0.59	**0.67**	0.59	0.43	0.44	0.57	0.58	0.59
		5000	0.63	**0.69**	0.63	0.44	0.51	0.60	0.58	0.62
		all	0.53	**0.69**	0.53	0.35	0.37	0.60	0.60	0.52

S2 Sparsity in Slide-seqV2 before and after applying Imputation

Table S2: Percentage of Zero Sparsity in the Slide-seqV2 datasets before (RAW) and after applying imputation methods.

SL No.	Top Genes	Dataset Name	RAW	MAGIC	KNN impute	Soft Impute	Simple Impute	scVI	gimVI	tangram
01.	2000	WT	93.32	0.29	93.32	0	0	0	0	0
		diabetes	93.53	0.45	93.53	0	0	0	0	0
		mouse	96.52	1.14	96.52	0	0	0	0	0
02.	5000	WT	93.90	0.52	93.90	0	0	0	0	0
		diabetes	93.85	0.60	93.85	0	0	0	0	0
03.	all	WT	94.02	0.76	94.02	0	0	0	0	0
		diabetes	93.73	0.96	93.73	0	0	0	0	0
		mouse	95.78	2.03	95.78	0	0	0	0	0

S3 Sparsity in XYZeq before and after applying Imputation

Table S3: Percentage of Zero Sparsity in the XYZeq datasets before (RAW) and after applying imputation methods.

SL No.	Top Genes	Dataset Name	RAW	MAGIC	KNN impute	Soft Impute	Simple Impute	scVI	gimVI	tangram
03.	2000	GSE 164430	97.8	14.3	97.8	0	0	0	0	0
		GSM 5009529	98.6	7.6	98.6	0	0	0	0	0
		GSM 5009539	98.6	6.8	98.6	0	0	0	0	0
03.	5000	GSE 164430	97.17	18.02	97.17	0	0	0	0	0
		GSM 5009529	98.26	8.18	98.26	0	0	0	0	0
		GSM 5009539	98.24	8.46	98.24	0	0	0	0	0
03.	all	GSE 164430	96.62	16.77	96.62	0	0	0	0	0
		GSM 5009529	97.87	8.86	97.87	0	0	0	0	0
		GSM 5009539	97.91	9.29	97.91	0	0	0	0	0

S4 Sparsity in sci-Space before and after applying Imputation

Table S4: Percentage of Zero Sparsity in the sci-Space datasets before (RAW) and after applying imputation methods.

SL No.	Top Genes	Dataset Name	RAW	MAGIC	KNN impute	Soft Impute	Simple Impute	scVI	gimVI	tangram
01.	2000	GSE 166692	86.61	3.99	86.61	0	0	0	0	0
02.	5000	GSE 166692	87.66	4.64	87.66	0	0	0	0	0
03.	all	GSE 166692	85.86	5.22	85.86	0	0	0	0	0

S5 Computational Performance of 'SpaMean-Impute' on SRT Technologies (excluding 10x Genomics Visium)

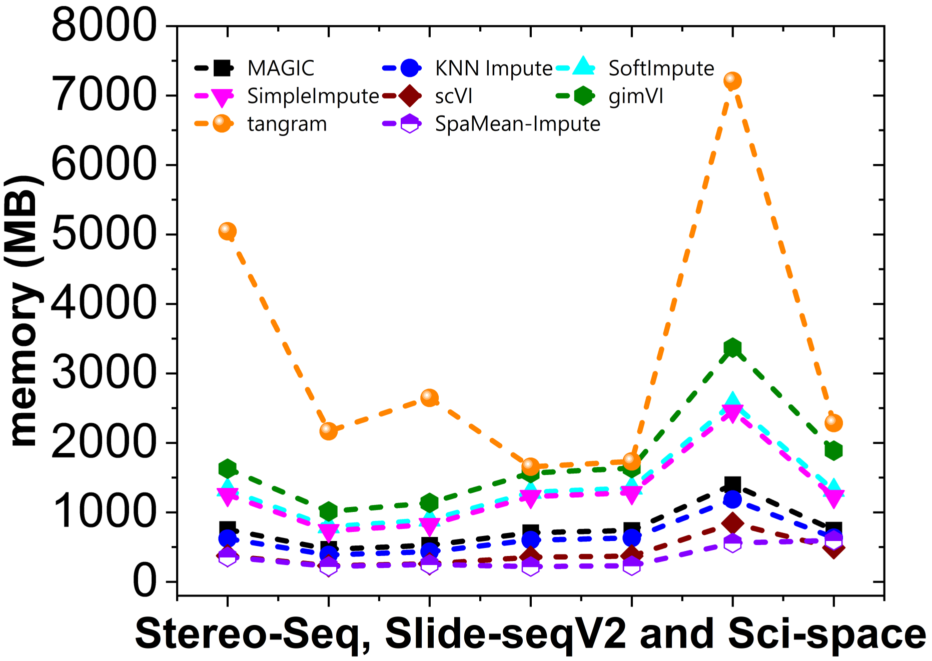

Fig. S1: Memory consumption comparison of 'SpaMean-Impute' with others for Stereo-seq, Slide-seqV2, sci-Space. 'tangram' consumes the highest memory as well, and 'SpaMean-Impute' consumes the lowest. Alt text: Memory consumption between the proposed method and other SOTA methods.

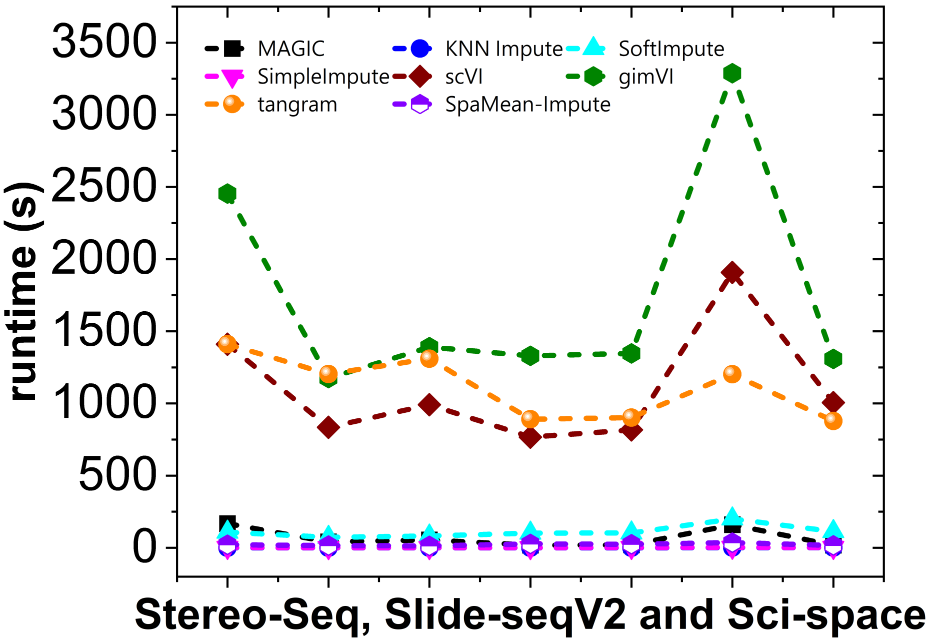

Fig. S2: Runtime comparison of 'SpaMean-Impute' with others for Stereo-seq, Slide-seqV2, sci-Space. 'gimVI' takes the highest runtime here as well, and 'SpaMean-Impute' takes the lowest. Alt text: Runtime comparison between proposed method and other SOTA methods.

S6 Sensitivity Analysis of 'SpaMean-Impute'

We have now performed a detailed sensitivity analysis of our method. We varied the K value from 3 to 15 and the dropout threshold value from 0.04 to 0.5 for all datasets and found the following results. The trend for two of the 10x Genomics Visium datasets can be observed in the following heatmaps in Figure S3. We can observe that the ARI value fluctuates from 0.21 to 0.375 for different combinations of K and dropout threshold value. Maximum ARI is achieved from 0.18 to 0.38 threshold, where the most consistent region (under the red rectangle region) is for K of 5 to 7. Similarly, we can observe that the NMI value fluctuates from 0.46 to 0.58 for different combinations of K and dropout threshold value. Maximum NMI is achieved from 0.18 to 0.48 threshold, where the most consistent region (under the red rectangle region) is for K of 3 to 5.

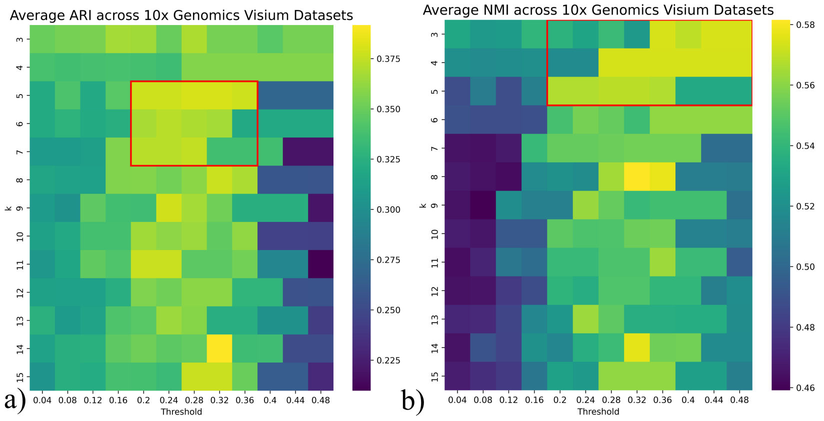

Fig. S3: Heatmap for sensitivity analysis varying the hyperparameters of SpaMean-Impute for two 10x Genomics Visium datasets for ARI and NMI. Alt text: Hyperparameter sensitivity of the proposed method.

We also performed this analysis for Slide-seqV2 as shown in Figure S4. We can again observe that the ARI value fluctuates from 0.15 to 0.21 for different combinations of K and dropout threshold value. Maximum ARI is achieved from 0.04 to 0.08 threshold, where the most consistent region (under the red rectangle region) is for K of 8 to 11. Similarly, we can observe that the NMI value fluctuates from 0.27 to 0.33 for different combinations of K and dropout threshold value. Maximum NMI is achieved from 0.04 to 0.12

threshold, where the most consistent region (under the red rectangle region) is for K of 6 to 9. This pattern across datasets indicates that our model's optimal hyperparameters tend to fall within narrow, consistent regions rather than being arbitrarily dispersed. By highlighting these "sweet spots" (red rectangles in Figures S3 and S4), we demonstrate that our results are robust to moderate changes in K and threshold, thereby strengthening the reliability of our findings and the generalizability of our method.

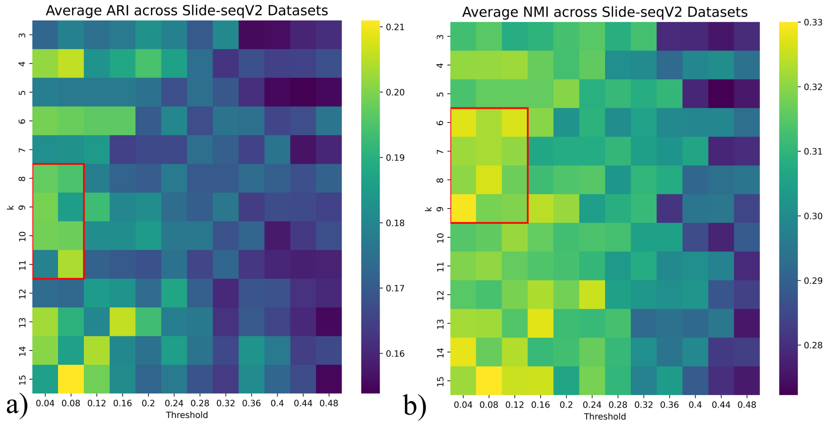

Fig. S4: Heatmap for sensitivity analysis varying the hyperparameters of SpaMean-Impute for Slide-seqV2 datasets for ARI and NMI. Alt text: Hyperparameter sensitivity of the proposed method.

S7 Performance of 'SpaMean-Impute' on recent SRT Datasets

Furthermore, we have applied our imputation framework to a new Visium dataset [1]. The results can be observed in Table S5. The downstream analysis indicates a consistent improvement after imputation.

Taken together, our results and literature review support the conclusion that, even with the most recent SRT technologies, data sparsity and related quality challenges remain prevalent [2, 3, 4, 5, 6, 7, 8, 1]. This justifies both the relevance of our study and the continued need for benchmarking imputation methods using well-established datasets with ground truth annotations.

Table S5: Performance of SpaMean-Impute on a new Visium dataset [1]

Technology	Dataset Name	Top Genes	Base ARI	Imputed ARI	Base NMI	Imputed NMI	Base AMI	Imputed AMI	Base HOMO	Imputed HOMO
Visium	Control02	all	0.63	**0.65**	0.80	**0.81**	0.79	**0.80**	0.80	**0.82**
	Control05	all	0.65	**0.67**	0.80	**0.81**	0.79	**0.80**	0.82	**0.83**
	Control06	all	0.62	**0.66**	0.77	**0.79**	0.77	**0.78**	0.76	**0.79**
	Control09	all	0.60	**0.63**	0.77	**0.79**	0.76	**0.78**	0.81	**0.84**

S8 Independent Test for True Signal Recovery for the proposed method

Our proposed algorithm works by averaging transcriptome profiles of cells that are not only transcriptionally similar but also spatially adjacent, under the assumption that neighboring cells with similar profiles are likely to belong to the same cell type. Potential circularity problem may arise under this assumption. Hence, we performed an independent test to demonstrate our model can truly recover the missing values. An independent test is performed that does not rely on tissue-domain alignment or UMAP clustering. Specifically, we artificially masked a proportion of non-zero values (1%, 3%, 5%, and 10%) in each dataset, applied our imputation algorithm, and then quantified how well the method could recover the original transcriptomic values. The summary of the independent test is shown in Table S6. This approach directly tests whether the algorithm genuinely recovers true signals rather than being optimized for clustering outcomes.

Across four different spatial transcriptomics technologies (Slide-seqV2, 10x Genomics Visium, Stereo-seq, and sci-Space), our algorithm demonstrated consistent recovery of masked non-zero values.

- **Imputation Rate:** Even at 10% masking, where data sparsity is high, our method still recovered a substantial fraction of the artificially masked values (e.g., 17–20% in Visium, ∼72–77% in Stereo-seq, and ∼22% in sci-Space). This shows robustness across a wide range of sparsity levels.

- **Correlation with Ground Truth:** We evaluated the imputed versus original data using three complementary measures: Pearson's (PCC), Spearman's (SRC), and Kendall's Tau (KTC). In every dataset, the

Table S6: Performance of the proposed imputation method under artificial masking across multiple spatial transcriptomics technologies. For each dataset, varying proportions of non-zero entries (1%, 3%, 5%, and 10%) were randomly masked to zero and then imputed. The table reports the total number of artificial zeros introduced, the number and percentage of zeros successfully imputed by our method, and the similarity between the original and imputed data as measured by Pearson correlation coefficient (PCC), Spearman rank correlation (SRC), and Kendall's Tau correlation (KTC).

**Technology**	**Dataset Name**	**Artificial Zeros (%)**	**Total Artificial Zeros**	**Imputed Zeros**	**Zeros Imputed (%)**	**PCC**	**SRC**	**KTC**
`Slide-seqV2`	diabetes	1	31959	9253	28.95	0.73	0.67	0.69
		3	95877	26090	27.21	0.70	0.66	0.67
		5	159796	39351	24.63	0.66	0.63	0.63
		10	319593	61516	19.25	0.60	0.58	0.57
	mouse	1	37447	4517	12.06	0.87	0.79	0.80
		3	112343	12469	11.10	0.84	0.76	0.77
		5	187238	18711	9.99	0.80	0.71	0.72
		10	374476	28594	7.64	0.73	0.61	0.62
`10x Genomics Visium`	151507	1	45487	10854	23.86	0.83	0.78	0.78
		3	135096	31202	23.10	0.80	0.76	0.76
		5	218406	45984	21.05	0.77	0.72	0.72
		10	414972	73021	17.60	0.71	0.65	0.64
	151673	1	45487	10968	24.11	0.83	0.77	0.78
		3	135096	31211	23.10	0.80	0.76	0.76
		5	218406	46270	21.19	0.76	0.72	0.71
		10	414972	72429	17.45	0.70	0.65	0.63
	151676	1	46478	12901	27.76	0.84	0.78	0.78
		3	138040	36409	26.38	0.82	0.76	0.75
		5	223165	54204	24.29	0.78	0.73	0.71
		10	424014	85753	20.22	0.73	0.66	0.64
`Stereo-seq`	DT2_D0	1	138905	120573	86.80	0.67	0.81	0.75
		3	416717	353649	84.87	0.65	0.80	0.74
		5	694529	564505	81.28	0.61	0.79	0.71
		10	1389058	999908	71.98	0.53	0.75	0.67
	FB2_D1	1	119344	109812	92.01	0.65	0.85	0.81
		3	358032	323440	90.34	0.63	0.84	0.79
		5	596720	518806	86.94	0.60	0.83	0.77
		10	1193440	918406	76.95	0.50	0.78	0.71
`sci-Space`	GSE166692	1	34706	11859	34.17	0.83	0.72	0.72
		3	104119	33483	32.16	0.81	0.71	0.70
		5	173532	49584	28.57	0.78	0.68	0.67
		10	347065	76063	21.92	0.72	0.61	0.59

correlations remained moderate to high, even as the masking fraction increased. For example:

  - In Slide-seqV2, PCC decreased from 0.73 at 1% masking to 0.60 at 10%, still indicating good recovery under heavy sparsity.
  - In Visium, PCC remained between 0.71–0.83, SRC ∼0.65–0.78, and KTC ∼0.63–0.78 across masking levels.
  - In Stereo-seq, despite the very large number of artificial zeros, the algorithm still achieved high recovery rates (70–90%) and solid correlation metrics.
  - In sci-Space, PCC stayed between 0.72–0.83, and SRC/KTC remained stable as well.

- As expected, the percentage of correctly imputed zeros and correlation coefficients gradually decreased as more data were masked (which is typical for any imputation algorithm), but our method consistently retained stronger recovery and correlations than random expectation, demonstrating true predictive capacity.

Together, these results show that our method is not merely making neighboring cells appear similar; rather, it accurately predicts missing transcriptomic values under a fully independent evaluation framework. Moreover, our method performed consistently across diverse platforms and tissue types, indicating that its performance is not limited to a specific dataset and maintained meaningful correlation across multiple statistical measures. This combination of recovery percentage, stability across masking levels, and multimetric correlations provides strong evidence that our approach represents a genuine advance over existing methods and is not an artifact of evaluation design.

References

[1] Barbara Di Marco, Javier Vázquez-Marín, Hannah Monyer, Lázaro Centanin, and Julieta Alfonso. Spatial transcriptomics map of the embryonic mouse brain–a tool to explore neurogenesis. *Biology Open*, 12(10):bio060151, 2023.

[2] Agus Salim, Dharmesh D Bhuva, Carissa Chen, Chin Wee Tan, Pengyi Yang, Melissa J Davis, and Jean YH Yang. Spanorm: spatially-aware normalization for spatial transcriptomics data. *Genome Biology*, 26(1):109, 2025.

[3] Yue You, Yuting Fu, Lanxiang Li, Zhongmin Zhang, Shikai Jia, Shihong Lu, Wenle Ren, Yifang Liu, Yang Xu, Xiaojing Liu, et al. Systematic comparison of sequencing-based spatial transcriptomic methods. *Nature Methods*, 21(9):1743–1754, 2024.

[4] Yu Zhao, Young Li, Ying He, Junqi Wu, Yi Liu, Xinxing Li, Zhaoxun Li, Qiaomei Yuan, Jialuo Li, Xinya Zhang, et al. Stereo-seq v2: Spatial mapping of total rna on ffpe sections with high resolution. *Cell*, 2025.

[5] Haoyu Wang, Peng Cheng, Juan Wang, Hongzhi Lv, Jie Han, Zhiyong Hou, Ren Xu, and Wei Chen. Advances in spatial transcriptomics and its application in the musculoskeletal system. *Bone Research*, 13(1):54, 2025.

[6] Stathis Megas, Anna Wilbrey-Clark, Aidan Maartens, Sarah A Teichmann, and Kerstin B Meyer. Spatial transcriptomics of the respiratory system. *Annual Review of Physiology*, 87, 2024.

[7] Nuray Sogunmez Erdogan and Deniz Eroglu. Sparse deconvolution of cell type medleys in spatial transcriptomics. *PLOS Computational Biology*, 21(6):e1013169, 2025.

[8] Sikta Das Adhikari, Jiaxin Yang, Jianrong Wang, and Yuehua Cui. Recent advances in spatially variable gene detection in spatial transcriptomics. *Computational and Structural Biotechnology Journal*, 23:883–891, 2024.
